# Long-Term Follow-Up of Patients with Axial Spondyloarthritis in Real-Life Setting: Results from Greece of the Multi-Country Registry Proof Study

**DOI:** 10.31138/mjr.251024.lot

**Published:** 2024-12-31

**Authors:** Gkikas Katsifis, Andreas Bounas, Anna Kandyli, Maria Koronaiou, Tina Antachopoulou, Antonios Kyriakakis, Dimos Patrikos

**Affiliations:** 1 Navl Hospital, Athens, Greece;; 2 OLYMPION Hospital-General Clinic of Patras, Patras, Greece;; 3 Private Practice, Athens, Greece;; 4 Medical Department, AbbVie Pharmaceuticals S.A., Neo Iraklio, Athens, Greece;; 5 “Metropolitan” General Hospital, Piraeus, Greece

**Keywords:** axial spondyloarthritis (axSpA), real-life, disease activity, disease progression

## Abstract

**Objectives:**

The aim of the present analysis was to describe the clinical and demographic characteristics of ankylosing spondylitis (AS) and non-radiographic axial spondyloarthritis (nr-axSpA) patients from Greece who were enrolled in the global PROOF study, and their longitudinal follow-up over 5 years to determine the impact of the disease on quality of life and patient-reported outcomes.

**Methods:**

PROOF was an observational study that enrolled recently diagnosed (<1 year) patients fulfilling the Assessment of SpondyloArthritis International Society classification criteria from rheumatology clinical practices from 29 countries across 6 different geographical regions.

**Results:**

Of the 100 Greek patients enrolled, 85 were classified based on local (investigator) evaluation of sacroiliac radiographs [AS: 56 (65.88%); nr-axSpA: 29 (34.12%)]. Higher prevalence of males in the AS (approximately 70%) and equal gender representation in the nr-axSpA patients were observed. There were variations in the clinical presentation, symptom duration, mean age at baseline, and HLA-B27 positivity between male and female patients. The majority of the patients were treated with TNF inhibitors from baseline to study end. Disease activity as well as patient-reported outcomes (functional index, quality of life, patient assessment of disease, and work productivity for employed patients) improved compared to baseline. Only 4 patients progressed to AS during the study.

**Conclusions:**

This analysis provides longitudinal data from the patients enrolled in the global PROOF study from Greece for comparative purposes. The data from the Greek cohort can highlight challenges in the management of the SpA patients.

## INTRODUCTION

Spondyloarthritis (SpA)- which can be axial (axSpA) or peripheral (pSpA) depending on the main skeletal involvement, is a diverse collection of inflammatory rheumatic illnesses with common clinical, biologic, and genetic features.^1^ Additionally, axSpA can be further classified in non-radiographic ax-SpA (nr-axSpA) and radiographic axSpA (r-axSpA), depending on the radiographic presence of structural damage in the sacroiliac joints or spine, respectively.^2^ SpA can range from early, undifferentiated forms to well-defined illnesses like ankylosing spondylitis (AS), which is considered the most severe axSpA subtype. The prevalence of axSpA varies widely due to genetic, racial, and environmental factors,^3,4^ with population-based studies reporting rates of 0.1–1.3% for AS and 0.3–1.9% for SpA.^5^ Data on AS incidence are limited, with variations influenced by ethnic background and HLA-B27 prevalence.^6, 7^ According to the ESORDIG study, the prevalence of SpA in Greece is on the lower range compared to other countries (0.49%).^8^ The prevalence of HLA-B27 in Greece was reported at 5.4% in 1979^9^ and 6% in 1989,10 aligning closely with other European Caucasian populations.^3^

AS does not always present with significant radiographic findings, namely sacroiliitis, which may take years to manifest, thus, making the establishment of diagnosis at early stages challenging.^2,5,11^ The modified New York criteria for AS, designed primarily for classification, focus solely on axial features and cannot reliably identify axSpA patients early in the disease course when radiographic sacroiliitis is absent.^12^ Hence, the Assessment in Spondylarthritis International Society (ASAS) were introduced to facilitate the identification of patients with SpA clinical features with or without radiographic evidence of sacroiliitis.^13,14^ Inflammatory chronic back pain (CBP) is the most prominent clinical symptom reported by 70–80% of axSpA patients, and is, therefore, relevant for classification and diagnosis.^5,13^ Despite recent improvements, the prompt diagnosis of axSpA remains challenging worldwide. The prompt identification of individuals who are likely to have axSpA among patients who present with CBP with age at onset ≤ 45 years and their subsequent referral to a rheumatologist for establishing a correct diagnosis are crucial to reduce the diagnostic delay of axSpA.^5^ ASAS classification criteria for referral for axSpA include patients younger than 45 years, with back pain for 3 months or more, sacroiliitis on imaging, plus one or more features of SpA or HLA-B27, plus two or more other features of SpA. A separate set of criteria is available for patients with peripheral rheumatological involvement without axial symptoms.^15^ The diagnostic delays may range from 0.67 to 8 years with a median delay of 2 to 6 years. Women with axSpA may face longer diagnostic delays due to differences in symptom patterns and disease severity.^16^ Additionally, despite having less spinal damage than men, women experience greater physical limitations and more severe symptoms such as scoliosis, bone loss, osteoarthritis, and compression.^17^ Although nr-axSpA affects equally men and women, it was hypothesized that axSpA was more prevalent in men; however, recent evidence contradicts this.

There are limited data on the long-term progression of nr-axSpA to AS as well as early-onset axSpA patients, including the factors that contribute to the emergence of radiographic sacroiliitis over time. These data are mainly derived from small, non-longitudinal studies, which lacked ASAS criteria.^18–22^ Following the introduction of ASAS criteria, it was realised that patients with nr-axSpA have heterogeneous clinical and genetic characteristics, thus, highlighting challenges in determining the prognosis to AS with the available clinical data.^22–24^ Further studies of long-term follow-up with an adequate sample size are, therefore, necessary. Evidence suggests that progression may manifest in early-onset axSpA patients, if followed-up adequately.^25^ Molto et al. performed a 10-year follow-up of early axSpA patients and reported that male sex, symptom duration >1.5 years, Axial Spondyloarthritis Disease Activity Score ≥2.1 and smoking (only in HLA-B27 positives) were associated with radiographically confirmed (based on modified New York criteria) progression to AS over 10 years’ follow-up.^26^ Further studies are needed to estimate the risk of progression and identify risk factors (clinical and demographic characteristics) for progression to AS in early-onset patients as well as the impact of the disease to implement timely risk-proportional overall therapeutic management approaches to patients who are at higher risk.^27^ Additionally, due to the impact of AS on employment status and quality of life (QoL), there is an unmet need to determine longitudinally both the risk for progression as well as the impact of the disease to the patients.^28–30^

PROOF [Patients With Axial Spondyloarthritis: Multi-country Registry of Clinical Characteristics (PROOF study), including radiographic progression and burden of disease over 5 years in real-life rheumatology clinical practice setting] was a large, multi-country, prospective, non-interventional study in recently diagnosed axSpA patients fulfilling the Assessment of SpondyloArthritis international Society classification that was conducted in 29 countries across 6 different geographical regions.^31–33^ The aim of this registry was to collect data that could contribute to the understanding of radiographic progression, clinical characteristics, and impact of the disease on quality of life and work productivity over time of nr-ax-SpA and AS patients over a 5-year follow-up. The results of the patients who were recruited in the PROOF study from Greece are presented in the current manuscript to compare with the data from global PROOF analysis and complement national data on the characteristics and management of afflicted patients.

## MATERIALS AND METHODS

### Study design

The clinical study was conducted in accordance with the ethical principles of the Declaration of Helsinki and all subsequent amendments, and applicable guidelines. The study was approved by the local ethics committees of each study site in accordance with local laws and regulations. The study eligibility criteria and assessments are detailed in Poddubnyy et al.^31^ PROOF enrolled adult patients who presented with CBP that could not be accounted for by prior diagnosis for at least 3 months prior to enrolment and had CBP onset before the age of 45 years. Exclusion criteria included prior diagnosis explaining CBP (among others it refers also to axSpA (AS or nr-axSpA) diagnosis if made > 12 months prior to Visit 1). Prior to inclusion, patients provided written informed consent. The first patient’s first visit in the Greek cohort occurred on 10 Jun 2014 and the last patient’s last visit on 30 Oct 2020.

### Study assessments-Statistical methodology

Treatment, procedures, and diagnostic methods followed physicians’ routine clinical practice. Other than baseline (Visit 1), the patients were followed up once yearly for overall 5 years (Visits 2–6).

Patients were classified at baseline as AS or nr--axSpA based on the evaluation of sacroiliitis on anteroposterior pelvic radiographs (r- or nr-axSpA) using modified New York criteria for AS first by a local reader, followed by a central reader.^34^ In case of a disagreement in the classification, the radiograph was evaluated by an adjudicator who was blinded to the previous assessments and set the final classification. In the cohort from Greece, only local reading was performed.

Clinical characteristics and disease burden as well as treatment patterns were assessed at baseline (Visit 1) and in 5 visits performed on an annual basis as per routine clinical practice (Visit 2–6) as previously described.^31,32,35^ Investigator confidence with the diagnosis of axSpA was ascertained on a numeric rating scale (0–10, with 0: not confident at all and 10: very confident) at enrolment. The statistical methodology has been described.^31,32,35^ All statistical analyses were carried out by SAS^®^.

## RESULTS

### Baseline Characteristics and Disposition

Overall, 100 patients were enrolled. Of those, diagnosis of AS or nr-axSpA based on radiography (local reading) was available for 85 patients altogether (56 and 29 patients respectively). The majority of the patients remained in the study throughout the 5 years (75 patients, 49 AS, and 26 nr-axSpA). There were minor differences between investigators’ judgment and local reading on diagnosis between AS or nr-axSpA based on radiography (**Supplementary Table 1**).

**Table 1.** Patient demographic and baseline clinical characteristics.

**Age (years)**	**Overall N=85**	**AS N=56**	**nr-axSpA N=29**	p-value^*^

Mean ±SD	43.0 ±11.1	43.9 ±11.4	41.3 ±10.3	0.383
Median	43.0	44.5	31.0	
IQR	36.0–49.0	36.5–49.5	35.0–48.0	
Min-Max	19.0–78.0	19.0–78.0	20.0–65.0	
Missing	0	0	0	
Range				
<30	6 (7.1)	3 (5.4)	3 (10.3)	
30–44	41 (48.2)	25 (44.6)	16 (55.2)	
45–49	17 (20.0)	14 (25.0)	3 (10.3)	
50–65	18 (21.2)	11 (19.6)	7 (24.1)	
>65	3 (3.5)	3 (5.4)	0 (.)	

**Gender**	**Overall**	**AS**	**nr-axSpA**	**p-value^†^**

Male	54 (63.5)	39 (69.6)	15 (51.7)	0.839
Female	31 (36.5)	17 (30.4)	14 (48.3)	
Missing	0	0	0	

* p-value of independent samples t-test,

† p-value of chi-square test

AS: Ankylosing spondylitis; IQR: Interquartile Range expressed as 25th–75th percentiles; nraxSpA: Non-radiographic axial spondyloarthritis; SD: Standard deviation.

The patient demographic characteristics are outlined in **Table 1**. There were no statistically significant differences in age and sex between AS and nr-axSpA patient groups. There was a slightly higher prevalence of males among AS patients (approximately 66% of AS patients) and an approximately equal percentage of males and females among nr-axSpA patients. The majority of patients per diagnosis fell in the 30–44 years age group, and nr-axSpA patients were younger than AS patients. The patients’ baseline clinical characteristics are shown in **Table 2**. There were differences in the interval between CBP onset, diagnosis and visit to physician/rheumatologist in the disease subgroups. For the overall population, the first visit to a physician due to back pain was at a median (Q1, Q3) of 30 (12; 102) months prior to the enrolment. Both the first referral to a rheumatologist and the assignment of diagnosis was made considerably later. The interval between the first visit to a physician and the first referral to a rheumatologist relative to Visit 1 was considerably lower for AS patients. In both disease groups, the majority of patients consulted a rheumatologist on their initiative. Sacroiliitis was confirmed primarily radiographically in both AS and nr-axSpA patients (>95% of patients). MRI was used in 27.6% of nr-axSpA and 17.9% of AS patients respectively. Based on radiography, the vast majority (approximately 80%) of nr-axSpA patients suffered from Grade 1 (82.8%–79.3%), whereas the vast majority of AS patients suffered from Grade 2–3 sacroiliitis in both sacroiliac joints. Additional SpA-related features were also evaluated at baseline. Testing for HLA-B27 test was not routinely performed in all patients at baseline and half of the AS patients tested positively for HLA-B27. A comparable percentage of AS and nr-axSpA patients suffered from psoriasis and arthritis. Regarding prior treatments, the majority of AS patients were administered NSAIDs (92.9%), The same pattern of past treatments was observed for nr-axSpA patients, though a lower percentage of nr-axSpA patients compared to AS patients was administered the aforementioned drug categories.

**Table 2.** Clinical characteristics at baseline.

	**Overall N=85**	**AS N=56**	**nr-axSpA N=29**

**Time from first visit at a physician due to back pain to Visit 1 in months**			

Mean ±SD	66.3 ±77.9	60.9 ±79.9	76.7 ±74.2
Median	30	26	58
IQR	12–102	11–85	15–115
Min – Max	1–420	1–420	6–300
Missing N	1	1	0

**Time from onset of chronic back pain to Visit 1 in months**			

Mean ±SD	88.0 ±105.3	100.2 ±115.2	64.4 ±79.5
Median	54	57.5	15
IQR	10–123	13–165	6–108
Min – Max	0–420	1–420	0–300
Missing N	0	0	0

**Time from first referral to a rheumatologist to Visit 1 in months**			

Mean ±SD	18.8 ±35.2	17.4 ±36.5	21.6 ±32.9
Median	4	3	6
IQR	2–12	2–11	2–15
Min – Max	0–211	0–211	0–118
Missing N	0	0	0

**Specialty of doctor doing the referral, N (%)**			

Dermatologist	4 (4.7)	0 (.)	4 (13.8)
Gastroenterologist	1 (1.2)	1 (1.8)	0 (.)
General Practitioner	5 (5.9)	1 (1.8)	4 (13.8)
Ophthalmologist	1 (1.2)	0 (.)	1 (3.4)
Orthopedist	20 (23.5)	15 (26.8)	5 (17.2)
Other	12 (14.1)	9 (16.1)	3 (10.3)
Initiative of the patient	41 (48.2)	29 (51.8)	12 (41.4)
Missing N	1	1	0

**Time from AS/nr-axSpA diagnosis to Visit 1 in months**	N=84	N=56	N=28

Mean ±SD	3 ± 3.7	3.2 ± 3.6	2.7 ± 3.8
Median	2	2	0.5
IQR	0–4	0–4	0–4.5
Min-Max	0–12	0–12	0–12

**Time from onset of chronic back pain to AS/nr-axSpA diagnosis in months**	N=84	N=56	N=28

Mean ±SD	85.3 ± 106.4	97 ± 115.7	61.8 ± 81.5
Median	47	54.5	9.5
IQR	3.5–135	6.5–164.5	2.5–113
Min – Max	0–420	0–420	0–300

***Sacroiliitis on imaging***	N=84	N=55	N=29

Active (acute) inflammation on MRI highly suggestive of sacroiliitis associated with SpA	16 (19.0)	3 (5.5)	13 (44.8)
Definite radiographic sacroiliitis according to modified New York criteria	66 (78.6)	50 (90.9)	16 (55.2)
Both	2 (2.4)	2 (3.6)	0 (0.0)

***Additional features***			

Good response to NSAIDs	32 (37.6)	24 (42.9)	8 (27.6)
Family history for SpA	4 (4.7)	4 (7.1)	0 (.)
HLA-B27 positive	31 (36.5)	26 (46.4)	5 (17.2)
Inflammatory back pain (IBP)	80(94.1)	53(94.6)	27 (93.1)
Psoriasis	20(23.5)	14(25.0)	6 (20.7)
Arthritis	65 (76.5)	44 (78.6)	21 (72.4)
Crohn’s disease or ulcerative colitis (IBD)	3 (3.5)	3 (5.4)	0 (.)
Enthesitis (heel)	57 (67.1)	44 (78.6)	13 (44.8)
Uveitis	4 (4.7)	1 (1.8)	3 (10.3)
Dactylitis	5 (5.9)	2 (3.6)	3 (10.3)
Elevated CRP	49 (57.6)	35 (62.5)	14 (48.3)

***Past Treatments***			

NSAIDs	70 (82.4)	52 (92.9)	18 (62.1)
DMARDs			
Methotrexate	16 (18.8)	13 (23.2)	3 (10.3)
Sulfasalazine	0 (.)	0 (.)	0 (.)
Other	4 (4.7)	3 (5.4)	1 (3.4)
Corticosteroids	16 (18.8)	10 (17.9)	6 (20.7)
Analgesics	30 (35.3)	24 (42.9)	6 (20.7)
TNF inhibitors	4 (4.7)	2 (3.6)	2 (6.9)
Months in total			
6	3 (3.5)	2 (3.6)	1 (3.4)
24	1 (1.2)	0 (.)	1 (3.4)

**Imaging examinations at baseline**	**Overall N=85**	**AS N=56**	**nr-axSpA N=29**

**Number of patients with X-ray performed**	83 (97.6)	55 (98.2)	28 (96.6)

Right Sacroiliac Joint			
***Grade 0***	3 (3.5)	2 (3.6)	1 (3.4)
***Grade 1***	25 (29.4)	1 (1.8)	24 (82.8)
***Grade 2***	21 (24.7)	18 (32.1)	3 (10.3)
***Grade 3***	24 (28.2)	24 (42.9)	0 (.)
***Grade 4***	10 (11.8)	10 (17.9)	0 (.)
Left Sacroiliac Joint			
***Grade 0***	2 (2.4)	0 (.)	2 (6.9)
***Grade 1***	24 (28.2)	1 (1.8)	23 (79.3)
***Grade 2***	25 (29.4)	22 (39.3)	3 (10.3)
***Grade 3***	22 (25.9)	22 (39.3)	0 (.)
***Grade 4***	10 (11.8)	10 (17.9)	0 (.)

***X-ray evaluation performed by: ***			

Rheumatologist	57 (67.1)	32 (57.1)	25 (86.2)
Radiologist	17 (20.0)	17 (30.4)	0 (.)
Rheumatologist + Radiologist	1 (1.2)	1 (1.8)	0 (.)
Missing N	10 (11.8)	6 (10.7)	4 (13.8)
**Number of patients with MRI performed**	18 (21.2)	10 (17.9)	8 (27.6)

AS: Ankylosing spondylitis; CRP: C-reactive protein; DMARD: Disease modifying antirheumatic drugs; HLA-B27: Human Leukocyte Antigen – B27; IBD: Inflammatory bowel disease; IQR: Interquartile Range expressed as 25th–75th percentiles; MRI: magnetic Resonance Imaging; NSAID: Non-steroidal anti-inflammatory drugs; nr-axSpA: Non-radiographic axial spondyloarthritis; SD: Standard deviation; TNF: Tumour Necrosis Factor.

* p-value of independent samples t-test,

† p-value of chi-square test.

### Concomitant diseases

There was a low incidence of concomitant diseases at baseline and throughout the study (**Table 3**). The most commonly reported concomitant diseases (reported between 10–20% of AS patients) were hyperlipidemia, hypertension, and depression in the AS group. In the nr-axSpA group, few patients were affected with concomitant diseases throughout the study.

**Table 3.** Comorbidities in the study population per visit.

**Comorbidities**	**N (%)**											

	**AS**						**nr-axSpA**					
	**Visit 1 N=56**	**Visit 2 N=56**	**Visit 3 N=55**	**Visit 4 N=53**	**Visit 5 N=53**	**Visit 6 N=49**	**Visit 1 N=29**	**Visit 2 N=29**	**Visit 3 N=27**	**Visit 4 N=27**	**Visit 5 N=27**	**Visit 6 N=26**

**CARDIOVASCULAR DISEASES**												

Hypertension	6 (10.7)	1 (1.8)	0 (.)	0 (.)	0 (.)	0 (.)	0 (.)	0 (.)	0 (.)	0 (.)	0 (.)	0 (.)
Coronary artery disease, including myocardial infarction	1 (1.8)	0 (.)	0 (.)	0 (.)	0 (.)	0 (.)	1 (3.4)	0 (.)	0 (.)	0 (.)	0 (.)	0 (.)
Stroke	1 (1.8)	0 (.)	0 (.)	0 (.)	0 (.)	0 (.)	0 (.)	0 (.)	0 (.)	0 (.)	0 (.)	0 (.)
Peripheral arterial Occlusive												
disease	1 (1.8)	0 (.)	0 (.)	0 (.)	0 (.)	0 (.)	0 (.)	0 (.)	0 (.)	0 (.)	0 (.)	0 (.)
Heart valve disease	1 (1.8)	0 (.)	0 (.)	0 (.)	0 (.)	0 (.)	0 (.)	0 (.)	0 (.)	0 (.)	0 (.)	0 (.)
Conduction disturbance	1 (1.8)	0 (.)	1 (1.8)	0 (.)	1 (1.9)	0 (.)	0 (.)	0 (.)	0 (.)	0 (.)	0 (.)	0 (.)
Chronic heart failure	1 (1.8)	0 (.)	0 (.)	0 (.)	0 (.)	0 (.)	0 (.)	0 (.)	0 (.)	0 (.)	1 (3.7)	0 (.)

**METABOLIC DISEASES**												

Hyperlipidaemia	9 (16.1)	0 (.)	0 (.)	0 (.)	1 (1.9)	0 (.)	2 (6.9)	0 (.)	0 (.)	0 (.)	0 (.)	0 (.)
Diabetes type I	1 (1.8)	–	–	–	–	–	0 (.)	–	–	–	–	–
Diabetes type II	1 (1.8)	0 (.)	0 (.)	0 (.)	0 (.)	0 (.)	1 (3.4)	0 (.)	0 (.)	0 (.)	0 (.)	0 (.)

**PULMONARY DISEASE**												

Chronic obstructive pulmonary disease	1 (1.8)	0 (.)	0 (.)	0 (.)	0 (.)	0 (.)	0 (.)	0 (.)	0 (.)	0 (.)	0 (.)	0 (.)
Restrictive pulmonary disease	1 (1.8)	0 (.)	0 (.)	0 (.)	0 (.)	0 (.)	0 (.)	0 (.)	0 (.)	0 (.)	0 (.)	0 (.)
	1 (1.8)	0 (.)	0 (.)	0 (.)	0 (.)	0 (.)		0 (.)	0 (.)	0 (.)	0 (.)	1 (3.8)
Pulmonary fibrosis							0 (.)					

**GASTROINTESTINAL**												

Gastric or duodenal ulcer	1 (1.8)	1 (1.8)	0 (.)	0 (.)	0 (.)	0 (.)	0 (.)	0 (.)	0 (.)	0 (.)	0 (.)	0 (.)
Gastritis	5 (8.9)	5 (8.9)	3 (5.5)	3 (5.7)	3 (5.7)	3 (6.1)	0 (.)	0 (.)	0 (.)	0 (.)	0 (.)	0 (.)
Diverticulitis	0 (.)	0 (.)	0 (.)	0 (.)	0 (.)	0 (.)	0 (.)	0 (.)	0 (.)	0 (.)	0 (.)	0 (.)

**MUSCULOSKELETAL**												

Degenerative spine disease	2 (3.6)	0 (.)	0 (.)	0 (.)	0 (.)	0 (.)	1 (3.4)	0 (.)	0 (.)	0 (.)	0 (.)	0 (.)
Osteoporosis	1 (1.8)	0 (.)	0 (.)	1 (1.9)	0 (.)	0 (.)	0 (.)	0 (.)	0 (.)	0 (.)	0 (.)	0 (.)
Fibromyalgia	3 (5.4)	0 (.)	0 (.)	0 (.)	0 (.)	0 (.)	1 (3.4)	0 (.)	0 (.)	0 (.)	0 (.)	0 (.)

**PSYCHIATRIC**												

Anxiety Depression	0 (.) 7 (12.5)	1 (1.8) 8 (14.3)	1 (1.8) 8 (14.5)	2 (3.8) 8 (15.1)	2 (3.8) 8 (15.1)	2 (4.1) 7 (14.3)	0 (.) 0 (.)	0 (.) 1 (3.4)	0 (.) 2 (7.4)	0 (.) 2 (7.4)	0 (.) 1 (3.7)	0 (.) 0 (.)

Cancer, including Lymphoma and Leukemia	0 (.)	0 (.)	0 (.)	0 (.)	0 (.)	0 (.)	0 (.)	0 (.)	0 (.)	1 (3.7)	0 (.)	0 (.)

AS: Ankylosing spondylitis; nr-axSpA: Non-radiographic axial spondyloarthritis.

Kidney diseases (kidney insufficiency or kidney amyloidosis) and Vasculitis (other autoimmune disease were not reported during the study.

### Disease Activity scores

Disease activity scores throughout the study are summarised in **Table 4**. All disease activity scores were indicative of high disease activity at baseline, and progressively decreased compared to baseline at each of the subsequent study visits.

**Table 4.** Disease activity scores (BASDAI, ASDAS CRP/ESR and PGA scores) per visit.

	**Overall**	**AS**	**nr-axSpA**	**Mean Difference (95% CI)**

**BASDAI**				

**Visit 1**	**N=85**	**N=56**	**N=29**	

Mean ±SD	5.5 ±1.9	6.0 ±1.7	4.7 ±1.9	1.30 (0.48, 2.11)
Median (IQR)	5.65 (4.0, 6.95)	5.85 (5.05, 7.1)	4 (3.05, 6.15)	
Min – Max	0.5–10	1.6–10	0.5–8.6	
Missing N	1	0	1	
Total N	84	56	28	

**Visit 2**	**N=85**	**N=56**	**N=29**	

Mean ±SD	2.3 ±1.5	2.6 ±1.5	1.7 ±1.3	0.85 (0.18, 1.51)
Median (IQR)	2 (1.7, 3.0)	2.3 (2.0, 3.3)	2 (0.8, 2.0)	
Min – Max	0–6.5	0–6.5	0–5.5	
Missing N	5	2	3	
Total N	80	54	26	

**Visit 3**	**N=82**	**N=55**	**N=27**	

Mean ±SD	1.9 ±1.4	2.1 ±1.5	1.5 ±1.0	0.62 (0.34, 1.21)
Median (IQR)	2 (0.8, 3.0)	2 (1.0, 3.1)	2 (0.6, 2.0)	
Min – Max	0–5.9	0–5.9	0–3.2	
Missing N	5	4	1	
Total N	77	51	26	

**Visit 4**	**N=80**	**N=53**	**N=27**	

Mean ±SD	2.0 ±1.5	2.4 ±1.7	1.4 ±0.8	1.01 (0.31, 1.71)
Median (IQR)	2 (1.0, 3.0)	2.2 (1.0, 3.6)	1.2 (1.0, 2.0)	
Min – Max	0–7.6	0–7.6	0–3.2	
Missing N	1	0	1	
Total N	79	53	26	

**Visit 5**	**N=80**	**N=53**	**N=27**	

Mean ±SD	1.4 ±1.0	1.7 ±1.1	1.0 ±0.7	0.64 (0.20, 1.07)
Median (IQR)	1 (0.8, 2.0)	1.6 (1.0, 2.3)	1 (0.6, 1.2)	
Min – Max	0–4.5	0–4.5	0–2.5	
Missing N	7	5	2	
Total N	73	48	25	
Visit 6	N=75	N=49	N=26	
Mean ±SD	1.7 ±1.2	1.9 ±1.2	1.1 ±1.2	0.75 (0.12, 1.38)
Median (IQR)	1.7 (1, 2.5)	1.9 (1, 2.5)	1 (0, 2)	
Min – Max	0–5.1	0–5.1	0–4.2	
Missing N	7	2	5	
Total N	68	47	21	

**ASDAS-CRP**				

**Visit 1**	**N=85**	**N=56**	**N=29**	

Mean ±SD	3.7 ±1.0	3.9 ±0.8	3.0 ±1.1	0.96 (0.41, 1.5)
Median (IQR)	3.9(3.1, 4.4)	4.0(3.5, 4.5)	3.1(2.3, 4.1)	
Min – Max	0.7–5.7	2.2–5.7	0.7–4.3	
Missing N	30	16	14	
Valid N	55	40	15	

**Visit 2**	**N=85**	**N=56**	**N=29**	

Mean ±SD	1.7 ±1.0	1.9 ±0.9	1.0 ±0.9	0.91 (0.35, 1.47)
Median (IQR)	1.7(0.8, 2.5)	1.9(1.4, 2.5)	0.9(0.4, 1.0)	
Min – Max	0.1–4.4	0.2–4.4	0.1–3.2	
Missing N	31	17	14	
Valid N	54	39	15	

**Visit 3**	**N=82**	**N=55**	**N=27**	

Mean ±SD	1.5 ±0.8	1.7 ±0.8	1.1 ±0.7	0.53 (0.03, 1.03)
Median (IQR)	1.4(1.0, 1.9)	1.5(1.3, 2.1)	1.1(0.6, 1.6)	
Min – Max	0.1–4.2	0.1–4.2	0.1–2.7	
Missing N	32	19	13	
Valid N	50	36	14	

**Visit 4**	**N=80**	**N=53**	**N=27**	

Mean ±SD	1.5 ±0.6	1.7 ±0.6	1.0 ±0.5	0.64 (0.29, 1.0)
Median (IQR)	1.5(1.0, 1.9)	1.7(1.4, 2.0)	0.9(0.6, 1.5)	
Min – Max	0.4–3.0	0.4–3.0	0.4–2.0	
Missing N	29	17	12	
Valid N	51	36	15	

**Visit 5**	**N=80**	**N=53**	**N=27**	

Mean ±SD	1.4 ±0.7	1.5 ±0.7	1.2 ±0.7	0.36 (−0.09, 0.8)
Median (IQR)	1.5(0.9, 1.9)	1.5(1.2, 1.9)	0.9(0.8, 1.4)	
Min – Max	0.1–3.0	0.1–2.8	0.4–3.0	
Missing N	33	20	13	
Valid N	47	33	14	

**Visit 6**	**N=75**	**N=49**	**N=26**	

Mean ±SD	1.6 ±0.8	1.7 ±0.7	1.3 ±0.9	0.39 (−0.14, 0.93)
Median (IQR)	1.5(1.0, 1.9)	1.6(1.3, 2.0)	1.0(0.6, 1.7)	
Min – Max	0.4–3.5	0.4–3.5	0.4–3.3	
Missing N	32	17	15	
Valid N	43	32	11	

**ASDAS-ESR**				

**Visit 1**	**N=85**	**N=56**	**N=29**	

Mean ±SD	3.4 ±0.6	3.6 ±0.6	3.1 ±0.5	0.56 (0.27, 0.85)
Median (IQR)	3.3 (3.0, 3.8)	3.7 (3.2, 4.1)	3.1 (2.9, 3.3)	
Min – Max	1.7–5.0	2.5–5.0	1.7–4.4	
Missing N	17	14	3	
Valid N	68	42	26	

**Visit 2**	**N=85**	**N=56**	**N=29**	

Mean ±SD	2.0 ±0.7	2.1 ±0.6	1.8 ±0.7	0.33 (0.1, 0.65)
Median (IQR)	2.1 (1.5, 2.4)	2.1 (1.8, 2.4)	1.7 (1.1, 2.4)	
Min – Max	0.6–3.6	0.8–3.6	0.6–3.3	
Missing N	7	4	3	
Valid N	78	52	26	

**Visit 3**	**N=82**	**N=55**	**N=27**	

Mean ±SD	1.8 ±0.7	1.9 ±0.6	1.6 ±0.7	0.30 (−0.02, 0.61)
Median (IQR)	1.9 (1.3, 2.3)	2.0 (1.3, 2.5)	1.8 (1.0, 2.2)	
Min – Max	0.4–3.7	0.8–3.7	0.4–2.6	
Missing N	5	4	1	
Valid N	77	51	26	

**Visit 4**	**N=80**	**N=53**	**N=27**	

Mean ±SD	1.8 ±0.6	1.9 ±0.6	1.4 ±0.5	0.49 (0.2, 0.77)
Median (IQR)	1.8 (1.3, 2.1)	2.0 (1.5, 2.4)	1.4 (0.9, 1.9)	
Min – Max	0.6–3.6	0.8–3.6	0.6–2.6	
Missing N	3	2	1	
Valid N	77	51	26	

**Visit 5**	**N=80**	**N=53**	**N=27**	

Mean ±SD	1.5 ±0.5	1.6 ±0.5	1.3 ±0.5	0.30 (0.05, 0.54)
Median (IQR)	1.5 (1.1, 1.8)	1.6 (1.3, 1.9)	1.4 (0.8, 1.6)	
Min – Max	0.6–2.6	0.7–2.6	0.6–2.2	
Missing N	7	5	2	
Valid N	73	48	25	

**Visit 6**	**N=75**	**N=49**	**N=26**	

Mean ±SD	1.5 ±0.6	1.7 ±0.6	1.3 ±0.6	0.34 (0.02, 0.66)
Median (IQR)	1.5 (1.1, 1.9)	1.6 (1.2, 2.1)	1.3 (1.0, 1.6)	
Min – Max	0.4–2.9	0.7–2.9	0.4–2.4	
Missing N	7	2	5	
Valid N	68	47	21	

**PGA scores**				

**Visit 1**	**N=85**	**N=56**	**N=29**	

Mean ±SD	5.27 ±2.25	5.59 ±2.32	4.66 ±2	0.93 (−0.08, 1.95)
Median (IQR)	5 (3, 8)	6 (4, 8)	4 (3, 6)	
Min – Max	0–9	0–9	1–9	
Total N	83	54	29	
Missing N	2	2	0	

**Visit 2**	**N=85**	**N=56**	**N=29**	

Mean ±SD	2.28 ±1.42	2.57 ±1.42	1.7 ±1.23	0.87 (0.23, 1.51)
Median (IQR)	2 (2, 3)	2 (2, 3)	2 (1, 2)	
Min – Max	0–6	0–6	0–5	
Total N	81	54	27	
Missing N	4	2	2	

**Visit 3**	**N=82**	**N=55**	**N=27**	

Mean ±SD	1.99 ±1.46	2.27 ±1.58	1.44 ±1.05	0.83 (0.16, 1.51)
Median (IQR)	2 (1, 3)	2 (1, 3)	2 (0, 2)	
Min – Max	0–7	0–7	0–3	
Total N	78	51	27	
Missing N	4	4	0	

**Visit 4**	**N=80**	**N=53**	**N=27**	

Mean ±SD	2.15 ±1.48	2.51 ±1.61	1.44 ±0.85	1.07 (0.51, 1.61)
Median (IQR)	2 (1, 3)	2 (2, 4)	1 (1, 2)	
Min – Max	0–7	0–7	0–3	
Total N	80	53	27	
Missing N	0	0	0	

**Visit 5**	**N=80**	**N=53**	**N=27**	

Mean ±SD	1.49 ±1.06	1.68 ±1.12	1.15 ±0.83	0.52 (0.02, 1.02)
Median (IQR)	1 (1, 2)	2 (1, 2)	1 (1, 2)	
Min – Max	0–4	0–4	0–3	
Total N	73	47	26	
Missing N	7	6	1	

**Visit 6**	**N=75**	**N=49**	**N=26**	

Mean ±SD	1.75 ±1.32	1.96 ±1.32	1.29 ±1.23	0.67 (−0.01, 1.35)
Median (IQR)	1.5 (1, 3)	2 (1, 3)	1 (0, 2)	
Min – Max	0–5	0–5	0–5	
Total N	68	47	21	
Missing N	7	2	5	

AS: Ankylosing spondylitis; ASDAS: Ankylosing spondylitis disease activity score; BASDAI: Bath Ankylosing Spondylitis Disease Activity Index; CI: Confidence interval; CRP: C-reactive protein; ESR: Erythrocyte sedimentation rate; IQR: Interquartile Range expressed as 25th–75th percentiles; nr-axSpA: Non-radiographic axial spondyloarthritis; PGA: Patient Global Assessment; SD: Standard deviation.

### Treatment administered during the study

At baseline, the majority of AS and nr-axSpA patients received TNF inhibitors (76.8% of AS patients and 65.5% of nr-axSpA patients), followed by NSAID(s) or methotrexate (**Table 5**). During the study, a comparable to baseline percentage of patients in either disease group continued to receive treatment with TNF inhibitors. There was a trend for decreased administration of NSAIDs during the study.

**Table 5.** Treatments administered during the study – Visits 1 to 6.

	**Overall N (%)**	**AS N (%)**	**nr-axSpa N (%)**

**Visit 1 (Baseline)**	**N=85**	**N=56**	**N=29**
NSAIDs	34 (40.0)	15 (26.8)	19 (65.5)
DMARDs			
Methotrexate	19 (22.4)	13 (23.2)	6 (20.7)
Sulfasalazine	1 (1.2)	1 (1.8)	0 (0.0)
Other	4 (4.7)	3 (5.4)	1 (3.4)
Corticosteroids	14 (16.5)	9 (16.1)	5 (17.2)
Analgesics	8 (9.4)	8 (14.3)	0 (0.0)
TNF inhibitors	62 (72.9)	43 (76.8)	19 (65.5)
**Visit 2**	**N=85**	**N=56**	**N=29**
NSAIDs	25 (29.4)	14 (25.0)	11 (37.9)
DMARDs			
Methotrexate	23 (27.1)	18 (32.1)	5 (17.2)
Sulfasalazine	0 (0.0)	0 (0.0)	0 (0.0)
Other	5 (5.9)	5 (8.9)	0 (0.0)
Corticosteroids	12 (14.1)	10 (17.9)	2 (6.9)
Analgesics	3 (3.5)	3 (5.4)	0 (0.0)
TNF inhibitors	61 (71.8)	38 (67.9)	23 (79.3)
**Visit 3**	**N=82**	**N=55**	**N=27**
NSAIDs	20 (24.4)	9 (16.4)	11 (40.7)
DMARDs			
Methotrexate	18 (22.0)	16 (29.1)	2 (7.4)
Sulfasalazine	0 (0.0)	0 (0.0)	0 (0.0)
Other	5 (6.1)	4 (7.3)	1 (3.7)
Corticosteroids	13 (15.9)	10 (18.2)	3 (11.1)
Analgesics	0 (0.0)	0 (0.0)	0 (0.0)
TNF inhibitors	60 (73.2)	38 (69.1)	22 (81.5)
**Visit 4**	**N=80**	**N=53**	**N=27**
NSAIDs	19 (23.8)	8 (15.1)	11 (40.7)
DMARDs			
Methotrexate	18 (22.5)	14 (26.4)	4 (14.8)
Sulfasalazine	1 (1.3)	0 (0.0)	1 (3.7)
Other	3 (3.8)	1 (1.9)	2 (7.4)
Corticosteroids	9 (11.3)	8 (15.1)	1 (3.7)
Analgesics	2 (2.5)	1 (1.9)	1 (3.7)
TNF inhibitors	57 (71.3)	37 (69.8)	20 (74.1)
**Visit 5**	**N=80**	**N=53**	**N=27**
NSAIDs	19 (23.8)	11 (20.8)	8 (29.6)
DMARDs			
Methotrexate	15 (18.8)	11 (20.8)	4 (14.8)
Sulfasalazine	0 (0.0)	0 (0.0)	0 (0.0)
Other	0 (0.0)	0 (0.0)	0 (0.0)
Corticosteroids	7 (8.8)	7 (13.2)	0 (0.0)
Analgesics	0 (0.0)	0 (0.0)	0 (0.0)
TNF inhibitors	53 (66.3)	34 (64.2)	19 (70.4)
**Visit 6**	**N=75**	**N=49**	**N=26**
NSAIDs	16 (21.3)	9 (18.4)	7 (26.9)
DMARDs			
Methotrexate	12 (16.0)	10 (20.4)	2 (7.7)
Sulfasalazine	0 (0.0)	0 (0.0)	0 (0.0)
Other	0 (0.0)	0 (0.0)	0 (0.0)
Corticosteroids	6 (8.0)	6 (12.2)	0 (0.0)
Analgesics	0 (0.0)	0 (0.0)	0 (0.0)
TNF inhibitors	52 (69.3)	34 (69.4)	18 (69.2)

AS: Ankylosing spondylitis; DMARD: disease-modifying antirheumatic drugs; nr-axSpA: Non-radiographic axial spondyloarthritis; NSAID: nonsteroidal anti-inflammatory drug; TNF: Tumour Necrosis Factor.

### Quality of Life

There was a trend for the norm-based SF-12v2 scores (NBS) and SF-6Dv2 utility score that were reported by nr-axSpA patients at baseline to be slightly higher or comparable to those reported by AS patients (**Table 6, Supplementary Table 2**). The mean (SD) SF-6Dv2 health utility scores baseline scores were comparable for AS and nr-axSpA subgroups. Across all SF-12v2 domains, there was an improvement compared to baseline that was sustained at all subsequent visits in both AS and nr-axSpA patients. Additionally, the mean NBS scores across all domains were higher for the nr-axSpA compared to AS patients.

**Table 6.** Quality of life [SF-12v2 norm-based scores (NBS)] per visit and health utility sccores (SF6Dv2).

**SF-12v2 NBS subscales, Mean (SD)**	**Visit 1**	**Visit 2**	**Visit 3**	**Visit 4**	**Visit 5**	**Visit 6**

**Physical Functioning NBS**						

**Total population**	**N=85** 39.01 ±6.41	**N=82** 48.33 ±7.82	**N=78** 48.48 ±7.31	**N=80** 49.58 ±7.91	**N=74** 51 ±7.66	**N=68** 50.23 ±8.03
**AS**	**N=56** 38.23 ±6.49	**N=55** 46.76 ±7.7	**N=51** 47.49 ±7.09	**N=53** 47.71 ±7.88	**N=48** 50.01 ±7.83	**N=47** 50.03 ±8.24
**nr-axSpA**	**N=29** 40.51 ±6.08	**N=27** 51.52 ±7.18	**N=27** 50.36 ±7.47	**N=27** 53.27 ±6.68	**N=26** 52.82 ±7.12	**N=21** 50.69 ±7.72
**Mean Difference (95% CI)**	−2.28 (−5.12, 0.57)	−4.76 (−8.28, −1.24)	−2.86 (−6.29, 0.56)	−5.57 (−9.09, −2.03)	−2.81 (−6.50, 0.87)	−0.66 (−4.90, 3.58)

**Role Physical NBS**						

**Total population**	**N=85** 38.3 ±6.49	**N=82** 48.79 ±6.27	**N=77** 50.04 ±7.24	**N=80** 50.32 ±7.27	**N=74** 52.73 ±5.48	**N=68** 50.87 ±6.59
**AS**	**N=56** 37.44 ±6.28	**N=55** 47.31 ±6.49	**N=50** 49.25 ±7.02	**N=53** 49.24 ±7.98	**N=48** 52.02 ±5.6	**N=47** 50.08 ±6.63
**nr-axSpA**	**N=29** 39.95 ±6.67	**N=27** 51.82 ±4.54	**N=27** 51.51 ±7.54	**N=27** 52.45 ±5.12	**N=26** 54.04 ±5.08	**N=21** 52.63 ±6.31
**Mean Difference (95% CI)**	−2.51 (−5.43, 0.40)	−4.51 (−7.29, −1.74)	−2.25 (−5.68, 1.17)	−3.21 (−6.14, −0.27)	−2.02 (−4.66, 0.61)	−2.55 (−5.97, 0.88)

**Bodily pain NBS**						

**Total population**	**N=84** 37.33 ±6.73	**N=82** 48.82 ±6.1	**N=77** 49.3 ±7.07	**N=79** 49.85 ±6.68	**N=74** 51.27 ±5.28	**N=68** 49.51 ±6.18
**AS**	**N=55** 36.74 ±6.27	**N=55** 47.56 ±6.03	**N=51** 48 ±7.4	**N=53** 48.37 ±7.07	**N=48** 50.97 ±5.1	**N=47** 48.52 ±5.79
**nr-axSpA**	**N=29** 38.45 ±7.52	**N=27** 51.38 ±5.49	**N=26** 51.83 ±5.67	**N=26** 52.87 ±4.59	**N=26** 51.83 ±5.67	**N=21** 51.72 ±6.59
**Mean Difference (95% CI)**	−1.70 (−4.78, 1.36)	−3.82 (−6.56, −1.08)	−3.83 (−7.13, −0.53)	−4.50 (−7.14, −1.86)	−0.87 (−3.44, 1.70)	−3.20 (−6.37, −0.03)

**General health NBS**						

**Total population**	**N=85** 37.91 ±9.99	**N=81** 53.05 ±8.29	**N=78** 54.25 ±8.11	**N=80** 53.74 ±8.45	**N=74** 56.03 ±6.91	**N=68** 54.33 ±8.06
**AS**	**N=56** 37.35 ±9.66	**N=55** 50.93 ±8.48	**N=51** 52.86 ±8.46	**N=53** 52.22 ±9.22	**N=48** 55.21 ±7.36	**N=47** 53.21 ±8.2
**nr-axSpA**	**N=29** 38.98 ±10.7	**N=26** 57.54 ±5.81	**N=27** 56.88 ±6.78	**N=27** 56.73 ±5.71	**N=26** 57.54 ±5.81	**N=21** 56.84 ±7.29
**Mean Difference (95% CI)**	−1.62 (−6.19, 2.93)	−6.06 (−9.83, −3.38)	−4.02 (−7.54, −0.50)	−4.52 (−7.86, −1.18)	−2.33 (−5.44, 0.78)	−3.63 (−7.79, 0.52)

**Vitality NBS**						

**Total population**	**N=83** 46.1 ±10.04	**N=82** 56.02 ±9.21	**N=78** 56.25 ±9.86	**N=80** 55.46 ±11.19	**N=73** 57.83 ±10.94	**N=68** 56.73 ±8.12
**AS**	**N=54** 46.15 ±9.86	**N=55** 54.07 ±8.85	**N=51** 53.7 ±8.65	**N=53** 52.22 ±11.54	**N=47** 54.09 ±10.83	**N=47** 54.51 ±7.62
**nr-axSpA**	**N=29** 46.01 ±10.55	**N=27** 59.99 ±8.77	**N=27** 61.09 ±10.33	**N=27** 61.82 ±7.12	**N=26** 64.58 ±7.45	**N=21** 61.71 ±7.05
**Mean Difference (95% CI)**	0.14 (−4.49, 4.77)	−5.92 (−10.04, −1.79)	−7.39 (−11.78, −3.00)	−9.59 (−13.77,−5.42)	−10.49 (−14.78, −6.19)	−7.20 (−11.11, −3.30)

**Social functioning NBS**						

**Total population**	**N=84** 37.95 ±7.53	**N=82** 48.77 ±6.44	**N=78** 50.17 ±7.19	**N=80** 49.34 ±6.8	**N=74** 51.73 ±6.25	**N=68** 49.97 ±6.85
**AS**	**N=55** 37.5 ±7.48	**N=55** 47.85 ±6.52	**N=51** 49.4 ±6.74	**N=53** 48.01 ±7.19	**N=48** 50.79 ±6.65	**N=47** 49.52 ±6.77
**nr-axSpA**	**N=29** 38.81 ±7.7	**N=27** 50.64 ±5.95	**N=27** 51.63 ±7.9	**N=27** 51.96 ±5.13	**N=26** 53.48 ±5.08	**N=21** 50.97 ±7.08
**Mean Difference (95% CI)**	−1.31 (−4.76, 2.14)	−2.80 (−5.76, 0.17)	−2.23 (−5.62, 1.17)	−3.95 (−7.05, −0.86)	−2.69 (−5.68, 0.29)	−1.45 (−5.05, 2.14)

**Role emotional NBS**						

**Total population**	**N=85** 34.21 ±7.65	**N=82** 46.08 ±8.06	**N=78** 48.15 ±8.61	**N=80** 48.16 ±7.62	**N=74** 50.38 ±6.28	**N=68** 48.41 ±7.7
**AS**	N=56 33.26 ±6.57	N=55 44.66 ±7.42	N=51 47.62 ±8.29	N=53 46.96 ±8.18	N=48 49.68 ±6.23	N=47 47.66 ±7.68
**nr-axSpA**	N=29 36.03 ±9.25	N=27 48.97 ±8.68	N=27 49.16 ±9.25	N=27 50.51 ±5.82	N=26 51.68 ±6.29	N=21 50.09 ±7.64
**Mean Difference (95% CI)**	−2.77 (−6.22, 0.68)	−4.31 (−8.23, −0.39)	−1.54 (−5.63, 2.25)	−3.54 (−7.06, −0.02)	−2.00 (−5.04, 1.03)	−2.44 (−6.46, 1.58)

**Mental health NBS**						

**Total population**	**N=84** 39.07 ±8.4	**N=82** 49.89 ±9.24	**N=78** 52.27 ±10.01	**N=80** 51.01 ±9.88	**N=74** 54.22 ±9.97	**N=68** 51.98 ±9.61
**AS**	**N=55** 38.34 ±7.72	**N=55** 47.83 ±9	**N=51** 50.01 ±9.31	**N=53** 48.07 ±9.6	**N=48** 51.31±10.08	**N=47** 50.66 ±10.04
**nr-axSpA**	**N=29** 40.47 ±9.54	**N=27** 54.08 ±8.42	**N=27** 56.54 ±10.05	**N=27** 56.77 ±7.76	**N=26** 59.58 ±7.26	**N=21** 54.92 ±8.01
**Mean Difference (95% CI)**	−2.13 (−5.96, 1.69)	−6.25 (−10.37, −2.13)	−6.52 (−11.07, −1.99)	−8.70 (−12.95, −4.45)	−8.27 (−12.33, −4.20)	−4.26 (−9.22, 0.70)

**SF6Dv2**						

**Total population**	**N=83** 0.576 ±0.075	**N=82** 0.717 ±0.128	**N=73** 0.758 ±0.15	**N=78** 0.754 ±0.153	**N=72** 0.812 ±0.152	**N=68** 0.76 ±0.146
**AS**	**N=54** 0.569 ±0.072	**N=55** 0.691 ±0.111	**N=48** 0.732 ±0.143	**N=52** 0.721 ±0.148	**N=46** 0.776±0.142	**N=47** 0.739 ±0.135
**nr-axSpA**	**N=29** 0.589 ±0.08	**N=27** 0.77 ±0.145	**N=25** 0.809 ±0.153	**N=26** 0.82 ±0.144	**N=26** 0.877±0.151	**N=21** 0.806 ±0.163
**Mean Difference (95% CI)**	−0.02 (−0.06, 0.02)	−0.08 (−0.14,−0.02)	−0.08 (−0.15, −0.01)	−0.10 (−0.17, −0.03)	−0.10 (−0.17, −0.03)	0.07 (−0.15, 0.02)

AS: Ankylosing spondylitis; BASFI: Bath Ankylosing Spondylitis Functional Index; CI: Confidence interval; IQR: Interquartile Range expressed as 25th–75th percentiles; NBS: norm-based scoring nr-axSpA: Non-radiographic axial spondyloarthritis; SD: Standard deviation; SF-12v2: Short Form-12v2.

The distribution of patients based on SF-12v2 levels demonstrated that, at baseline, the majority of AS patients performed below or well below in both the physical and mental components relative to the general population and in the mental component relative to the arthritis population (**Supplementary Table 3**). The same pattern was observed for nr-axSpA patients at baseline, except for the mental component relative to the arthritis population, whereby an approximately equal percentage of nr-axSpA patients responded same, below, or well below. The percentage of patients that performed same or better to the general population and the arthritis population in both the physical and mental components increased during the study in both subgroups.

### Physical Function

Physical function throughout the study was determined BASFI (**Supplementary Table 4**). The mean (SD) BASFI values were 5.5 (±1.9) and 3.4 (±1.5) for the AS and nr-axSpA groups at baseline, respectively. At the end of the study the corresponding scores were 1.9 (±1.3) and 1.0 ( ±1.1) for the AS and nr-axSpA patients respectively. Compared to baseline, there was a decrease in BASFI scores, indicating improvement in physical function throughout the study.

### Work Productivity

Approximately half of AS (44.6%) and nr-axSpA (48.3%) patients were employed at Visit 1 and this percentage increased in both groups, particularly in the nr-ax-SpA group, until Visit 3 in patients with available data (**Supplementary Table 5**).

Among employed patients, there was a trend for the mean WPAI-SHP absenteeism scores (% work missed due to problem) and the percentage of patients with “no absenteeism” to increase in both subgroups compared to baseline during the study (**Supplementary Table 6**). There was, also, a trend for the mean WPAI-SHP presenteeism, productivity, and activity impairment scores as well as the percentage of patients with “no presenteeism”, “productivity loss” and “activity impairment” to decrease in both subgroups compared to baseline during the study decreased (**Supplementary Table 7, 8, 9**). These trends should be interpreted with caution due to the progressively low number of patients per subgroup during the study.

### Progression to AS

**Supplementary Figure 1.**
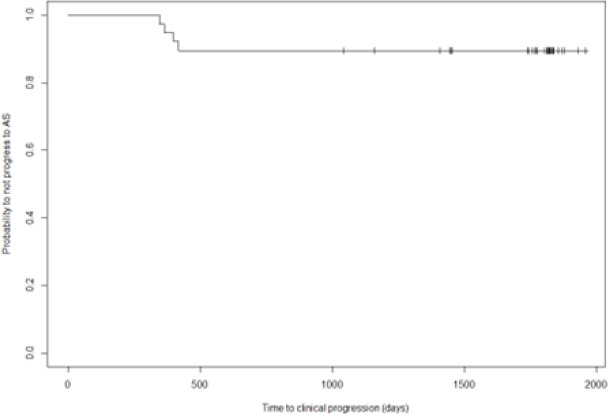
Kaplan – Meier curve for the time to progression to AS.

Over the duration of the study, 4 patients progressed from nr-axSpA to AS (**Supplementary Figure 1, Supplementary Table 10**).

### Analysis by sex

The analyses by sex should be treated with caution due to the small sample of women. Men with AS were statistically significantly more frequently HLA-B27 positive (59% vs 13.6%, p=0.001), and suffered from inflammatory back pain compared to women with AS (97.4% vs 68.2%, p=0.001) (**Supplementary Table 11**). High CRP levels were, also, statistically significantly more frequently reported in nr-asSpA men compared to women (71.4% vs 26.7%, p=0.040). By sex, women were non-statistically significantly older than men among both AS and nr-axSpA patients. Symptom duration was comparable between male and female AS patients, however, among nr-axSpA patients, women had almost twice as long symptom duration compared to men.

By TNF inhibitor use and sex, women with AS that used TNF inhibitors were older than men in the same group (48.7 vs 41.3, p=0.031) and were less frequently HLA-B27 positive (22.2% vs 61.8%, p=0.034) (**Supplementary Table 12**). High CRP levels were statistically significantly more frequently reported in men with nr-axSpA who were administered TNF inhibitors compared to women (70.0% vs 11.1%, p=0.009).

Disease activity by sex decreased from baseline during the study for men and women in both disease subgroups by ASDAS CRP/ESR (**Supplementary Table 13**) and BASDAI/BASFI score (**Supplementary Table 14**). The majority of men and women who were diagnosed with AS (79.4% and 64.3% of AS patients respectively) presented very high disease activity at baseline. The majority of men and women who were diagnosed with nr-axSpA (61.5% and 64.3% of nr-axSpA patients respectively) presented high disease activity at baseline. Interestingly, 14.3% of female patients with nr-axSpA had inactive disease at baseline. At the end of the study, approximately 50% of male and female AS patients had low disease activity. Among nr-axSpA patients, at the end of the study, 50% of females attained low disease activity and 69.2% of males had inactive disease. There was a trend for a progressively higher percentage of male patients in both disease subgroups to attain inactive disease during the study compared to baseline. There was also a trend for a progressively higher percentage of female patients in both disease subgroups to attain low disease activity during the study compared to baseline.

A higher percentage of nr-axSpA male patients compared to females attained inactive disease at Visit 6 (69.2% vs 25.0%, p=0.049). The mean (SD) BASDAI score results were statistically significantly lower in male compared to female nr-axSpA patients at Visit 6. No statistically significant differences were observed based on sex in BASFI results. Overall, due to the progressively lower number of patients with data availability towards the end of the study, these results should be interpreted with caution.

## DISCUSSION

The PROOF study contributed information on the clinical presentation and impact of AS and nr-axSpA on recently diagnosed (≤1 year) patients who fulfilled the ASAS classification criteria longitudinally up to 5 years. The results from the analysis of the cohort from Greece are in line with the global results. Thus, the majority of patients who presented with CBP were diagnosed with AS (∼65%) at baseline. There were some similarities and variations in the demographic and clinical baseline characteristics between the Greek cohort and the global PROOF study. Compared to the global PROOF study results, the Greek cohort, regardless of diagnosis, was older (mean age: 36.3 years)^36^ and nr-axSpA patients were slightly younger than AS patients.^36^ Similarly to the global PROOF results, among nr-axSpA patients approximately 50% were males. Only a few patients presented with concomitant diseases in the Greek cohort throughout the study, which is probably attributed to the age range of the patients. The most frequently reported concomitant diseases were hyper-lipidaemia, hypertension, and depression in both subgroups from Greece. The incidence of depression was low, and it was more frequently reported in AS patients. A cluster analysis of studies on axSpA confirmed that the two most common comorbidities were hypertension and depression.^37^ Most patients from Greece were non-smokers throughout the study, which could have contributed to the reduced prevalence of cardiovascular diseases over the 5-year follow-up. Comorbidities contribute to the burden of disease via interfering with disease activity, functional, and work disability, and mortality.^38^ A recent cluster analysis on axSpA patients demonstrated that some comorbidities, namely depression-anxiety, and fibromyalgia-inflammatory bowel syndrome, were associated with poorer health outcomes and increased axSpA severity.^37^ The effect of comorbidity burden on patient-reported outcome assessments could not be ruled out.^39^ Thus, the higher incidence of depression in AS patients could potentially contribute to the general trend for lower scores in QoL assessments compared to nr-axSpA patients at baseline and throughout the study. Both the mean duration of CBP from onset to diagnosis for the Greek cohort (85.3 months) and the longer duration to diagnosis for the AS compared to nr-axSpA subgroup were consistent with the global PROOF results (mean symptom duration 82.1 months).^36^ Real-life studies confirm that there is a considerable delay between symptom onset, referral to doctor and diagnosis for SpA (up to 6.7 years), which in turn are related to greater functional impairment, QoL deterioration and higher healthcare costs.^40–42^ Per disease subgroup, patients with AS had a higher occurrence of enthesitis (78.6% vs. 44.8%), psoriasis (25% vs. 20.7%) and elevated C-reactive protein (CRP) (62.5% vs. 48.3%) compared to nr-axSpA patients at baseline. In the contrary, nr-axSpA patients presented higher percentual of uveitis (10.3% vs. 1.8%, respectively) and dactylitis (10.3% vs. 3.6%, respectively) than AS patients. These observations are in agreement with the global PROOF results.^36^

The diagnosis of SpA is based on a careful evaluation of both imaging and clinical features.^43^ Radiography was the main imaging approach in both AS and nr-axSpA subgroups, and MRI was used in nr-axSpA patients mainly for diagnostic purposes. The introduction of MRI of the axial skeleton in the diagnostic and classification approaches in axSpA has substantially improved early diagnosis. There were some differences between investigator diagnosis and local radiography reading, which may contribute to the diagnostic delays that patients with SpA face in routine clinical practice and in turn highlight the importance of increasing awareness among patients with persistent CBP and healthcare professionals for prompt referral. In addition to inflammatory activity assessment (active inflammation in the sacroiliac joints and to a further extent in the spine on MRI, elevated CRP), the evaluation of extra-articular manifestations is, also, crucial, because these factors can determine optimal treatment suggestions in SpA patients.^43^ The implementation of a checklist that will include the thorough assessment of clinical signs and symptoms, including changes in their severity, in addition to clinical examination at regular patient follow-up visits and imaging can contribute to the prompt diagnosis of SpA in clinical practice.^44^

The baseline disease activity scores, functional ability (BASFI), and PGA parameters were worse for the Greek cohort compared to the baseline global PROOF results.^36^ At baseline, the disease scores for the Greek cohort were consistent with high disease activity, which was reflected on the patient-reported outcomes (work productivity, functional ability, PGA and QoL assessments) that were investigated in this study. Throughout the study (Visit 1–6) the majority of Greek patients in either subgroup was administered TNF inhibitors followed by NSAIDs. TNF inhibitors contribute to the improvement of all SpA manifestations, CRP levels, and MRI-detectable inflammation in the sacroiliac joints or spine and provenly slow radiographic progression, although it is proposed that AS women have a double risk of failing TNF inhibitor treatment compared to men.^45^ The small sample of AS women in the current study precluded reaching conclusions on real-life treatment patterns. Overall, for the entire Greek cohort treatment with TNF inhibitors was effective and by the end of the follow-up period, both disease subgroups had optimal disease control (low disease activity or inactive disease), which was also in line with the improvement in patient-reported outcomes physical function, patient global assessment of disease activity, quality of life, and work impairment in both AS and nr-axSpA patients throughout the study duration.^35^ As opposed to the Greek cohort analysis, the use of TNF inhibitors from the 1^st^ year of the study was significantly lower in the global PROOF results. NSAIDS were the most frequently administered treatment, followed by DMARDs, and progressively increasing use of TNF inhibitors was observed from Visit 1 to Visit 6 in the global analysis.^35^ The potential differences in the prescribed treatments between the Greek cohort and the global PROOF results could reflect both market access and local prescription patterns. The choice of treatments could, also, account for the observed differences in disease activity patterns between the Greek cohort data and the global PROOF results.^35^

Based on the global study results, central X-ray assessment revealed that 16% of nr-axSpA patients progressed to r-axSpA within 5 years with a mean time to progression of 2.4 years. In the Greek cohort, only 4 of 38 nr-axS-pA patients (10.52%) progressed to AS and the time to progression was not reached. The percentage of patients who progressed to AS was comparable to the global study results^36^ and literature data, which suggest that up to 20% of nr-axSpA progressed to AS over 8 years of follow-up.^22–24^ QoL, based on SF12v2, was impaired early on in the disease course. QoL assessments scores remained approximately comparable between nr-axSpA and AS patients longitudinally. Although inflammatory biomarker levels were higher and physical activity was more impaired in AS compared to nr-axSpA patients, the patient-perceived impact of the disease on productivity, activity impairment, and quality of life in nr-axSpA patients was equally grave to AS patients at baseline and longitudinally, which complies with the available literature data comparing nr-axSpA vs axSpA patients.^30,46^

Similarly to the global PROOF results and literature evidence, there was a higher prevalence of men vs women among AS patients (approximately two-thirds of AS patients) and HLA-B27 positivity was statistically significantly higher in male compared to female AS patients,^16,31^ whereas both men and women were equally affected with nr-axSpA.^36,47^ The known association between HLA-B27 and AS pathology accounts for the statistically significantly higher HLA-B27 positivity in male compared to female AS patients. Disease activity and BASFI scores were approximately comparable in women and men in both disease subgroups in the Greek cohort at baseline, indicating that women were equally impacted by the disease. The analysis of progression from nr-axSpA to AS over the 5-year-period of the global PROOF demonstrated that male sex correlated with progression to AS.^32^ Among nr-axSpA Greek patients, women had almost twice as long symptom duration compared to men, which is in line with literature data on diagnostic delays in women with SpA.^16^ The latter can be attributed to the different pattern and severity of clinical symptoms, but, also, to the slower radiographic progression in women who are afflicted with the disease.^17,48^

One of the inherent limitations of long-term follow-up studies is the potential for missing data, the risk for loss of follow-up, and attrition bias. Particularly for the extrapolation of the data to Greek patients, the limited sample size may be an additional limitation. The study was conducted prior to the latest update of the corresponding ASAS-EULAR recommendations and the therapeutic armamentarium of the SpA patients has expanded significantly since then.^49^ Due to imbalance in the sex of the enrolled patients, conclusions from the subgroup analysis by sex should be handled with caution. Central reading of the radiographs was not implemented for the Greek cohort, thus progression to AS could not be determined as per the protocol-specified criteria. Despite the aforementioned limitations, the conduct of the study under conditions of routine clinical practice in addition to the long-term follow-up outweigh the aforementioned limitations. The consecutive enrolment of patients limits the potential patient selection bias. The collection of patient-reported outcomes across a range of everyday aspects is, also, one of the strengths of the current study, as up-to-date guidelines emphasize the importance of patient-reported outcome collection, along with laboratory tests, clinical evaluation, and imaging for disease monitoring.

## CONCLUSION

This analysis of the national data corroborates the global RPOOF study results. It also contributes evidence on a national basis in Greece on the demographics, clinical presentation, and disease impact of AS and nr-axSpA on patients’ lives longitudinally. The observed differences and similarities to global data relate to local treatment patterns, diagnostic approaches, and the clinical characteristics of the enrolled patients. It is, also, worth mentioning that the treatment armamentarium for AS is currently broader compared to the treatments that were marketed when the PROOF study was conducted.^49^ The analysis of the data from the Greek cohort adds to the limited national epidemiological data and highlights routine clinical practice challenges in the management of SpA patients in Greece and unmet needs.

## Figures and Tables

**Supplementary Table 1.** Comparison of investigator’s judgment versus local reading for diagnosis.

**N (%)**	**Local Reading**			
**Investigator clinical judgement**	**Total N=85**	**AS N=56**	**nr-axSpA N=29**	p-value^*^
AS	53	45	8	0.648
nr – axSpA	32	11	21	

* p-value of McNemar’s test for the equivalency of diagnoses

AS: Ankylosing spondylitis; nr-axSpA: Non-radiographic axial spondyloarthritis.

**Supplementary Table 2.** Distribution of SF-12v2 scores per visit.

**SF-12v2 subscales, Mean (SD)**	**Visit 1**	**Visit 2**	**Visit 3**	**Visit 4**	**Visit 5**	**Visit 6**

**Physical Functioning Total population**						
					
	**N=85** 42.65 ±20.35	**N=82** 72.26 ±24.85	**N=78** 72.76 ±23.21	**N=80** 76.25 ±25.13	**N=74** 80.74 ±24.32	**N=68** 78.31 ±25.52
**AS**	**N=56** 40.18 ±20.6	**N=55** 67.27 ±24.47	**N=51** 69.61 ±22.54	**N=53** 70.28 ±25.03	**N=48** 77.6 ±24.86	**N=47** 77.66 ±26.19
**nr-axSpA**	**N=29** 47.41 ±19.3	**N=27** 82.41 ±22.8	**N=27** 78.7 ±23.72	**N=27** 87.96 ±21.22	**N=26** 86.54 ±22.62	**N=21** 79.76 ±24.52
**Mean Difference (95% CI)**	−7.24 (−16.28, 1.80)	**−15.13 (−26.3, −3.94)**	−9.1 (−20.0, 1.78)	**−17.68 (−28.90, −7.00)**	−8.9 (−20.6, 2.77)	−2.1 (−15.56, 11.36)
**Role Physical**						
**Total population**	**N=85** 43.38 ±19.15	**N=82** 74.39 ±18.52	**N=77** 78.08 ±21.38	**N=80** 78.91 ±21.48	**N=74** 86.03 ±16.18	**N=68** 80.51 ±19.48
**AS**	**N=56** 40.85 ±18.54	**N=55** 70 ±19.18	**N=50** 75.75 ±20.73	**N=53** 75.71 ±23.57	**N=48** 83.93 ±16.55	**N=47** 78.19 ±19.59
**nr-axSpA**	**N=29** 48.28 ±19.69	**N=27** 83.33 ±13.43	**N=27** 82.41 ±22.27	**N=27** 85.19 ±15.13	**N=26** 89.9 ±15.01	**N=21** 85.71 ±18.66
**Mean Difference (95% CI)**	−7.43 (−16.04, 1.19)	**−13.33 (−21.53, −5.14)**	−6.66 (−16.78, 3.47)	−9.48 (−19.42, 0.47)	−5.97 (−13.76, 1.80)	−7.52 (−17.64, 2.53)
**Bodily Pain**						
**Total population**	**N=84** 43.45 ±18.66	**N=82** 75.3 ±16.89	**N=77** 76.62 ±19.59	**N=79** 78.16 ±18.5	**N=74** 82.09 ±14.64	**N=68** 77.21 ±17.13
**AS**	**N=55** 41.82 ±17.38	**N=55** 71.82 ±16.7	**N=51** 73.04 ±20.52	**N=53** 74.06 ±19.59	**N=48** 81.25 ±14.12	**N=47** 74.47 ±16.06
**nr-axSpA**	**N=29** 46.55 ±20.84	**N=27** 82.41 ±15.21	**N=26** 83.65 ±15.72	**N=26** 86.54 ±12.71	**N=26** 83.65 ±15.72	**N=21** 83.33 ±18.26
**Mean Difference (95% CI)**	−4.73 (−13.24, 3.77)	−10.59 (−18.18, −3.00)	**−10.61 (−19.76, −1.47)**	**−12.48 (−20.90, −4.07)**	−2.40 (−9.54, 4.73)	**−8.87 (−17.65, −0.08)**
**General health**						
**Total population**	**N=85** 35.24 ±25.13	**N=81** 73.33 ±20.86	**N=78** 76.35 ±20.4	**N=80** 75.06 ±21.25	**N=74** 80.81 ±17.38	**N=68** 76.54 ±20.26
**AS**	N=56 33.84 ±24.29	N=55 68 ±21.34	N=51 72.84 ±21.29	N=53 71.23 ±23.2	N=48 78.75 ±18.52	N=47 73.72 ±20.63
**nr-axSpA**	N=29 37.93 ±26.91	N=26 84.62 ±14.62	N=27 82.96 ±17.06	N=27 82.59 ±14.37	N=26 84.62 ±14.62	N=21 82.86 ±18.34
**Mean Difference (95% CI)**	−4.09 (−15.56, 7.38)	**−16.62 (−24.72, −8.51)**	**−10.12 (−19.00, −1.26)**	**−11.37 (−19.77,−2.96)**	−5.87 (−13.7, 1.97)	−9.13 (−19.60, 1.33)
**Vitality**						
**Total population**	N=83 42.47 ±25.52	N=82 67.68 ±23.4	N=78 68.27 ±25.06	N=80 66.25 ±28.43	N=73 72.26 ±27.81	N=68 69.49 ±20.65
**AS**	N=54 42.59 ±25.06	N=55 62.73 ±22.5	N=51 61.76 ±22	N=53 58.02 ±29.32	N=47 62.77 ±27.52	N=47 63.83 ±19.37
**nr-axSpA**	N=29 42.24 ±26.81	N=27 77.78 ±22.29	N=27 80.56 ±26.25	N=27 82.41 ±18.1	N=26 89.42 ±18.94	N=21 82.14 ±17.93
**Mean Difference**	0.35	−15.05	**−18.79**	**−24.39**	**−26.66**	**−18.31**
**(95% CI)**	(−11.41, 12.11)	(−25.54, −4.56)	**(−29.95, −7.63)**	**(−36.68, −12.09)**	**(−38.76, −14.55)**	**(−28.05, −8.57)**
**Social functioning**						
**Total population**	N=84 46.73 ±21.18	N=82 77.13 ±18.09	N=78 81.09 ±20.22	N=80 78.75 ±19.12	N=74 85.47 ±17.56	N=68 80.51 ±19.24
**AS**	**N=55** 45.45 ±21.02	**N=55** 74.55 ±18.31	**N=51** 78.92 ±18.96	**N=53** 75 ±20.22	**N=48** 82.81 ±18.71	**N=47** 79.26 ±19.02
**nr-axSpA**	**N=29** 49.14 ±21.63	**N=27** 82.41 ±16.72	**N=27** 85.19 ±22.21	**N=27** 86.11 ±14.43	**N=26** 90.38 ±14.28	**N=21** 83.33 ±19.9
**Mean Difference (95% CI)**	−3.68 (−13.38, 6.00)	−7.86 (−16.19, 0.47)	−6.26 (−15.80, 3.27)	−11.11 (−19.81, −2.41)	−7.57 (−15.97, 0.82)	−4.08 (−14.19, 6.03)
**Role emotional**						
**Total population**	**N=85** 46.91 ±18.39	**N=82** 75.46 ±19.39	**N=78** 80.45 ±20.7	**N=80** 80.47 ±18.32	**N=74** 85.81 ±15.11	**N=68** 81.07 ±18.51
**AS**	**N=56** 44.64 ±15.79	**N=55** 72.05 ±17.83	**N=51** 79.17 ±19.95	**N=53** 77.59 ±19.67	**N=48** 84.11 ±14.98	**N=47** 79.26 ±18.48
**nr-axSpA**	**N=29** 51.29 ±22.25	**N=27** 82.41 ±20.88	**N=27** 82.87 ±22.24	**N=27** 86.11 ±14.01	**N=26** 88.94 ±15.14	**N=21** 85.12 ±18.38
**Mean Difference** **(95% CI)**	−6.65 (−14.94, 1.64)	−10.36 (19.19, −1.53)	4.94 (−13.54, 6.14)	−8.52 (−16.13, −0.90)	−4.83 (−12.12, 2.47)	−5.86 (−15.53, 3.81)
**Mental health**						
**Total population**	**N=84** 45.23 ±18.31	**N=82** 68.8 ±20.14	**N=78** 73.99 ±21.81	**N=80** 71.24 ±21.53	**N=74** 78.22 ±21.71	**N=68** 73.35 ±20.93
**AS**	**N=55** 43.62 ±16.83	**N=55** 64.32 ±19.61	**N=51** 69.06 ±20.3	**N=53** 64.84 ±20.92	**N=48** 71.9 ±21.97	**N=47** 70.48 ±21.87
**nr-axSpA**	**N=29** 48.28 ±20.79	**N=27** 77.93 ±18.34	**N=27** 83.29 ±21.89	**N=27** 83.8 ±16.92	**N=26** 89.9 ±15.82	**N=21** 79.76 ±17.44
**Mean Difference (95% CI)**	−4.65 (−13.00, 3.69)	−13.60 (−22.59, −4.63)	−14.23 (−24.11, −4.34)	**−18.96 (−28.22, −9.70)**	**−18.00 (−26.86, −9.14)**	−9.28 (−20.10. 1.53)
**Physical component summary**						
**Total population**	**N=84** 39.69 ±6.29	**N=82** 50.23 ±5.94	**N=78** 50.24 ±6.43	**N=80** 51.13 ±6.96	**N=74** 52.5 ±5.37	**N=68** 51.39 ±6.14
**AS**	**N=55** 39.12 ±6.55	**N=55** 48.78 ±5.89	**N=51** 49.28 ±6.53	**N=53** 49.88 ±7.66	**N=48** 52.21 ±5.73	**N=47** 50.81 ±6.01
**nr-axSpA**	**N=29** 40.78 ±5.71	**N=27** 53.2 ±4.93	**N=27** 52.04 ±5.94	**N=27** 53.58 ±4.52	**N=26** 53.04 ±4.69	**N=21** 52.69 ±6.39
**Mean Difference (95% CI)**	−1.67 (−4.53, 1.19)	**−4.42 (−7.04, −1.8)**	−2.75 (−5.76, 0.25)	**−3.70 (−6.42, −0.98)**	−0.83 (−3.45, 1.78)	−1.88 (−5.10, 1.33)
**Mental component summary**						
**Total population**	**N=84** 39.04 ±7.86	**N=82** 49.93 ±8.24	**N=78** 52.17 ±8.96	**N=80** 50.81 ±8.28	**N=74** 53.74 ±8.12	**N=68** 51.67 ±7.64
**AS**	**N=55** 38.5 ±7.35	**N=55** 48.31 ±7.39	**N=51** 50.51 ±7.5	**N=53** 48.48 ±8.17	**N=48** 51.26 ±7.82	**N=47** 50.31 ±7.51
**nr-axSpA**	**N=29** 40.08 ±8.8	**N=27** 53.23 ±9.01	**N=27** 55.3 ±10.68	**N=27** 55.38 ±6.47	**N=26** 58.31 ±6.65	**N=21** 54.69 ±7.19
**Mean Difference (95% CI)**	−1.58 (−5.18, 2.01)	**−4.92 (−8.96, −0.89)**	**−4.78(−8.91, −0.65)**	**−6.90(−10.50, −3.30)**	**−7.04 (−10.65, −3.43)**	**−4.38 (−8.25, −0.51**

AS: Ankylosing spondylitis; CI: Confidence interval; IQR: Interquartile Range expressed as 25th–75th percentiles; nr-axSpA: Non-radiographic axial spondyloarthritis; SD: Standard deviation.

**Supplementary Table 3.** Distribution of patients according to the levels of SF-12.

N (% )^*^		**Visit 1**	**Visit 2**	**Visit 3**	**Visit 4**	**Visit 5**	**Visit 6**

**Total Population**							

**General population**	Same or Better	10 (11.9)	67 (81.7)	62 (79.5)	63 (78.8)	67 (90.5)	55 (80.9)
**Physical Component**	Below	36 (42.9)	12 (14.6)	14 (17.9)	14 (17.5)	6 (8.1)	9 (13.2)
	Well Below	38 (45.2)	3 (3.7)	2 (2.6)	3 (3.8)	1 (1.4)	4 (5.9)
**General population**	Same or Better	16 (19.0)	61 (74.4)	62 (79.5)	54 (67.5)	58 (78.4)	53 (77.9)
**Mental Component**	Below	31 (36.9)	17 (20.7)	11 (14.1)	21 (26.3)	14 (18.9)	11 (16.2)
	Well Below	37 (44.0)	4 (4.9)	5 (6.4)	5 (6.3)	2 (2.7)	4 (5.9)
**General Arthritis population**	Same or Better	56 (66.7)	79 (117.9)	77 (116.7)	78 (132.2)	73 (121.7)	67 (98.5)
**Physical Component**	Below	19 (22.6)	3 (4.5)	1 (1.5)	1 (1.7)	1 (1.7)	1 (1.5)
	Well Below	9 (10.7)	0 (0.0)	0 (0.0)	1 (1.7)	0 (0.0)	0 (0.0)
**General Arthritis population**	Same or Better	20 (23.8)	64 (95.5)	65 (98.5)	57 (96.6)	59 (98.3)	57 (83.8)
**Mental Component**	Below	31 (36.9)	15 (22.4)	8 (12.1)	20 (33.9)	13 (21.7)	9 (13.2)
	Well Below	33 (39.3)	3 (4.5)	5 (7.6)	3 (5.1)	2 (3.3)	2 (2.9)

**AS**							

**General population**	Same or Better	6 (10.9)	41 (74.5)	38 (74.5)	37 (69.8)	42 (87.5)	37 (78.7)
**Physical Component**	Below	21 (38.2)	11 (20.0)	11 (21.6)	13 (24.5)	5 (10.4)	7 (14.9)
	Well Below	28 (50.9)	3 (5.5)	2 (3.9)	3 (5.7)	1 (2.1)	3 (6.4)
**General population**	Same or Better	9 (16.4)	36 (65.5)	38 (74.5)	29 (54.7)	33 (68.8)	35 (74.5)
**Mental Component**	Below	19 (34.5)	16 (29.1)	10 (19.6)	20 (37.7)	14 (29.2)	8 (17.0)
	Well Below	27 (49.1)	3 (5.5)	3 (5.9)	4 (7.5)	1 (2.1)	4 (8.5)
**General Arthritis population**	Same or Better	33 (60.0)	52 (123.8)	50 (119.0)	51 (150.0)	47 (134.3)	46 (117.9)
**Physical Component**	Below	15 (27.3)	3 (7.1)	1 (2.4)	1 (2.9)	1 (2.9)	1 (2.6)
	Well Below	7 (12.7)	0 (0.0)	0 (0.0)	1 (2.9)	0 (0.0)	0 (0.0)
**General Arthritis population**	Same or Better	11 (20.0)	39 (92.9)	41 (97.6)	32 (94.1)	34 (97.1)	38 (97.4)
**Mental Component**	Below	20 (36.4)	14 (33.3)	7 (16.7)	19 (55.9)	13 (37.1)	7 (17.9)
	Well Below	24 (43.6)	2 (4.8)	3 (7.1)	2 (5.9)	1 (2.9)	2 (5.1)

**nr-axSpA**							

**General population**	Same or Better	4 (13.8)	26 (96.3)	24 (88.9)	26 (96.3)	25 (96.2)	18 (85.7)
**Physical Component**	Below	15 (51.7)	1 (3.7)	3 (11.1)	1 (3.7)	1 (3.8)	2 (9.5)
	Well Below	10 (34.5)	0 (0.0)	0 (0.0)	0 (0.0)	0 (0.0)	1 (4.8)
**General population**	Same or Better	7 (24.1)	25 (92.6)	24 (88.9)	25 (92.6)	25 (96.2)	18 (85.7)
**Mental Component**	Below	12 (41.4)	1 (3.7)	1 (3.7)	1 (3.7)	0 (0.0)	3 (14.3)
	Well Below	10 (34.5)	1 (3.7)	2 (7.4)	1 (3.7)	1 (3.8)	0 (0.0)
**General Arthritis population**	Same or Better	23 (79.3)	27 (100.0)	27 (100.0)	27 (100.0)	26 (100.0)	21 (100.0)
**Physical Component**	Below	4 (13.8)	0 (0.0)	0 (0.0)	0 (0.0)	0 (0.0)	0 (0.0)
	Well Below	2 (6.9)	0 (0.0)	0 (0.0)	0 (0.0)	0 (0.0)	0 (0.0)
**General Arthritis population**	Same or Better	9 (31.0)	25 (92.6)	24 (88.9)	25 (92.6)	25 (96.2)	19 (90.5)
**Mental Component**	Below	11 (37.9)	1 (3.7)	1 (3.7)	1 (3.7)	0 (0.0)	2 (9.5)
	Well Below	9 (31.0)	1 (3.7)	2 (7.4)	1 (3.7)	1 (3.8)	0 (0.0)

AS: Ankylosing spondylitis; nr-axSpA: Non-radiographic axial spondyloarthritis,

* Percentages based on available data.

**Supplementary Table 4.** BASFI scores per visit.

	**Overall**	**AS**	**nr-axSpA**	**Mean Difference (95% CI)**

**Visit 1**	**N=85**	**N=56**	**N=29**	

Mean ±SD	4.8 ±2.0	5.5 ±1.9	3.4 ±1.5	2.12 (1.30, 2.95)
Median (IQR)	4.7 (3.2, 6.3)	5.4 (4.5, 7.0)	3.2 (2.8, 4.2)	
Min – Max	0.2–9.6	0.9–9.6	0.2–6.8	
Missing N	2	1	1	
Valid N	83	55	28	

**Visit 2**	**N=85**	**N=56**	**N=29**	

Mean ±SD	2.1 ±1.4	2.5 ±1.5	1.4 ±1.1	1.06 (0.41, 1.7)
Median (IQR)	2.0 (1.3, 3.0)	2.3 (1.8, 3.0)	2.0 (0.4, 2.0)	
Min – Max	0.0–6.5	0.0–6.5	0.0–3.5	
Missing N	5	2	3	
Valid N	80	54	26	

**Visit 3**	**N=82**	**N=55**	**N=27**	

Mean ±SD	1.8 ±1.3	2.1 ±1.4	1.3 ±0.9	0.80 (0.18, 1.4)
Median (IQR)	1.8 (0.7, 2.9)	2.0 (1.1, 3.0)	1.5 (0.3, 2.0)	
Min – Max	0.0–5.7	0.0–5.7	0.0–3.0	
Missing N	6	5	1	
Valid N	76	50	26	

**Visit 4**	**N=80**	**N=53**	**N=27**	

Mean ±SD	1.9 ±1.7	2.4 ±1.9	1.0 ±0.7	1.39 (0.8, 2.0)
Median (IQR)	1.6 (0.7, 3.0)	2.0 (1.0, 3.8)	1.0 (0.3, 1.6)	
Min – Max	0.0–7.8	0.0–7.8	0.0–2.0	
Missing N	3	2	1	
Valid N	77	51	26	

**Visit 5**	**N=80**	**N=53**	**N=27**	

Mean ±SD	1.4 ±1.3	1.7 ±1.4	0.8 ±0.8	0.86 (0.27, 1.5)
Median (IQR)	1.0 (0.5, 2.0)	1.6 (1.0, 2.5)	1.0 (0.2, 1.0)	
Min – Max	0.0–7.7	0.0–7.7	0.0–3.2	
Missing N	8	6	2	
Valid N	72	47	25	

**Visit 6**	**N=75**	**N=49**	**N=26**	

Mean ±SD	1.7 ±1.3	1.9 ±1.3	1.0 ±1.1	0.90 (0.24, 1.6)
Median (IQR)	1.5 (1.0, 2.4)	1.9 (1.0, 2.8)	1.0 (0.0, 2.0)	
Min – Max	0.0–5.8	0.0–5.8	0.0–3.7	
Missing N	9	3	6	
Valid N	66	46	20	

AS: Ankylosing spondylitis; BASFI: Bath Ankylosing Spondylitis Functional Index; CI: Confidence interval; IQR: Interquartile Range expressed as 25th–75th percentiles; nr-axSpA: Non-radiographic axial spondyloarthritis; SD: Standard deviation.

**Supplementary Table 5.** Employment Status of patients by visit.

	**Overall**	**AS**	**nr-axSpa**

**Visit 1**	N=85	N=56	N=29
**Employed**	39 (45.9)	25 (44.6)	14 (48.3)
**Unemployed**	46(54.1)	30 (53.6)	15 (51.7)
**Missing**	1(1.2)	1 (1.8)	0 (.)
**Visit 2**	N=85	N=56	N=29
**Employed**	48 (56.5)	32 (57.1)	16 (55.2)
**Unemployed**	33 (38.8)	22 (39.3)	11 (37.9)
**Missing**	4 (4.7)	2 (3.6)	2 (6.9)
**Visit 3**	N=82	N=55	N=27
**Employed**	48 (58.5)	29 (52.7)	19 (70.4)
**Unemployed**	30 (36.6)	22 (40.0)	8 (29.6)
**Missing**	4 (4.9)	4 (7.3)	0 (0.0)
**Visit 4**	N=80	N=53	N=27
**Employed**	50 (62.5)	31 (58.5)	19 (70.4)
**Unemployed**	30 (37.5)	22 (41.5)	8 (29.6)
**Missing**	0 (0.0)	0 (0.0)	0 (0.0)
**Visit 5**	N=80	N=53	N=27
**Employed**	40 (50.0)	23 (43.4)	17 (63.0)
**Unemployed**	34 (42.5)	25 (47.2)	9 (33.3)
**Missing**	6 (7.5)	5 (9.4)	1 (3.7)
**Visit 6**	N=75	N=49	N=26
**Employed**	28 (37.3)	16 (32.7)	12 (46.2)
**Unemployed**	40 (53.3)	31 (63.3)	9 (34.6)
**Missing**	7 (9.3)	2 (4.1)	5 (19.2)

AS: Ankylosing spondylitis; nr-axSpA: Non-radiographic axial spondyloarthritis.

**Supplementary Table 6.** WPAI-SHP Absenteeism scores (Percent work time missed due to problem–For Employed Patients only).

	**Overall**	**AS**	**nr-axSpa**	**Mean Difference (95% CI)**

**Visit 1**	N=39	N=25	N=14	
**No Absenteeism**	28 (71.8)	15 (60.0)	13 (92.9)	
**Absenteeism**	10 (25.6)	10 (40.0)	0 (0.0)	
***Missing***	**1 (2.6)**	**0 (0.0)**	**1 (7.1)**	
***Mean ±SD***	19.5 ±25.6	19.5 ±25.6	–	–
***Median (IQR) ***	4 (3, 50)	4 (3, 50)	–	
***Min – Max***	3–60	3–60	–	
**Visit 2**	**N=48**	**N=32**	**N=16**	
**No Absenteeism**	**36 (75.0)**	**24 (75.0)**	**12 (75.0)**	
**Absenteeism**	**7 (14.6)**	**5 (15.6)**	**2 (12.5)**	
***Missing***	**5 (10.4)**	**3 (9.4)**	**2 (12.5)**	
***Mean ±SD***	44.3 ±40.2	56.2 ±42.4	14.5 ±3.5	41.7 (−10.7, 94.1)
***Median (IQR) ***	17 (14, 100)	50 (17, 100)	14.5 (12, 17)	
***Min – Max***	12–100	14–100	12–17	
**Visit 3**	**N=48**	**N=29**	**N=19**	
**No Absenteeism**	**41 (85.4)**	**26 (89.7)**	**15 (78.9)**	
**Absenteeism**	**4 (8.3)**	**3 (10.3)**	**1 (5.3)**	
***Missing***	**3 (6.3)**	**0 (0.0)**	**3 (15.8)**	
***Mean ±SD***	25.8 ±18.9	24 ±22.7	31 ±.	−7 (−119.9, 105.9)
***Median (IQR) ***	22.5 (11, 40.5)	14 (8, 50)	31 (31, 31)	
***Min – Max***	8–50	8–50	31–31	
**Visit 4**	**N=50**	**N=31**	**N=19**	
**No Absenteeism**	**41 (85.4)**	**26 (89.7)**	**15 (78.9)**	
**Absenteeism**	**4 (8.3)**	**3 (10.3)**	**1 (5.3)**	
***Missing***	**3 (6.3)**	**0 (0.0)**	**3 (15.8)**	
***Mean ±SD***	45.5 ±45.3	34.6 ±40.9	100 ±.	−64.4(−189.8, 59.0)
***Median (IQR) ***	29.5 (7, 100)	9 (7, 50)	100 (100, 100)	
***Min – Max***	7–100	7–100	100–100	
**Visit 5**	**N=40**	**N=23**	**N=17**	
**No Absenteeism**	**31 (77.5)**	**14 (60.9)**	**17 (100.0)**	
**Absenteeism**	**9 (22.5)**	**9 (39.1)**	**0 (0.0)**	
***Missing***	**0 (0.0)**	**0 (0.0)**	**0 (0.0)**	
***Mean ±SD***	41.2 ±30.3	41.2 ±30.3	–	–
***Median (IQR) ***	50 (7, 50)	50 (7, 50)	–	
***Min – Max***	7–100	7–100	–	
**Visit 6**	**N=28**	**N=16**	**N=12**	
**No Absenteeism**	**18 (64.3)**	**11 (68.8)**	**7 (58.3)**	
**Absenteeism**	**5 (17.9)**	**5 (31.3)**	**0 (0.0)**	
***Missing***	**5 (17.9)**	**0 (0.0)**	**5 (41.7)**	
***Mean ±SD***	50 ±0	50 ±0	–	–
***Median (IQR) ***	50 (50, 50)	50 (50, 50)	–	
***Min – Max***	50–50	50–50	–	

AS: Ankylosing spondylitis; CI: Confidence interval; IQR: Interquartile Range expressed as 25th–75th percentiles; nr-axSpA: Non-radiographic axial spondyloarthritis; SD: Standard deviation; WPAI-SHP: Work Productivity and Activity Impairment Specific Health Problem.

**Supplementary Table 7.** WPAI-SHP Presenteeism scores (Percent impairment while working due to problem–For Employed Patients only).

	**Overall**	**AS**	**nr-axSpA**	**Mean Difference (95% CI)**

**Visit 1**	**N=39**	**N=25**	**N=14**	
**No Presenteeism**	**2 (5.1)**	**1 (4.0)**	**1 (7.1)**	
**Presenteeism**	**37 (94.9)**	**24 (96.0)**	**13 (92.9)**	
***Missing***	**0 (0.0)**	**0 (0.0)**	**0 (0.0)**	
***Mean ±SD***	48.4 ±23.3	56.7 ±22.2	33.1 ±17	23.6 (9.2, 38.0)
***Median (IQR) ***	40 (30, 70)	55 (40, 80)	30 (20, 40)	
***Min – Max***	20–90	20–90	20–80	
**Visit 2**	**N=48**	**N=32**	**N=16**	
**No Presenteeism**	**11 (22.9)**	**7 (21.9)**	**4 (25.0)**	
**Presenteeism**	**37 (77.1)**	**25 (78.1)**	**12 (75.0)**	
***Missing***	**0 (0.0)**	**0 (0.0)**	**0 (0.0)**	
***Mean ±SD***	24.3 ±12.4	25.6 ±14.5	21.7 ±5.8	3.9 (−2.8, 10.71)
***Median (IQR) ***	20 (20, 30)	20 (20, 30)	20 (20, 25)	
***Min – Max***	10–70	10–70	10–30	
**Visit 3**	**N=48**	**N=29**	**N=19**	
**No Presenteeism**	**13 (27.1)**	**7 (24.1)**	**6 (31.6)**	
**Presenteeism**	**35 (72.9)**	**22 (75.9)**	**13 (68.4)**	
***Missing***	**0 (0.0)**	**0 (0.0)**	**0 (0.0)**	
***Mean ±SD***	21.4 ±9.7	23.2 ±10.9	18.5 ±6.9	4.7 (−1.4, 10.8)
***Median (IQR) ***	20 (10, 30)	20 (10, 30)	20 (10, 20)	
***Min – Max***	10–40	10–40	10–30	
**Visit 4**	**N=50**	**N=31**	**N=19**	
**No Presenteeism**	**11 (22.0)**	**8 (25.8)**	**3 (15.8)**	
**Presenteeism**	**39 (78.0)**	**23 (74.2)**	**16 (84.2)**	
***Missing***	**0 (0.0)**	**0 (0.0)**	**0 (0.0)**	
***Mean ±SD***	23.3 ±13	28.3 ±14	16.3 ±7.2	12.0 (5.0, 19.0)
***Median (IQR) ***	20 (10, 30)	30 (20, 40)	15 (10, 20)	
***Min – Max***	10–50	10–50	10–30	
**Visit 5**	**N=40**	**N=23**	**N=17**	
**No Presenteeism**	**11 (27.5)**	**5 (21.7)**	**6 (35.3)**	
**Presenteeism**	**29 (72.5)**	**18 (78.3)**	**11 (64.7)**	
***Missing***	**0 (0.0)**	**0 (0.0)**	**0 (0.0)**	
***Mean ±SD***	15.5 ±6.3	17.2 ±6.7	12.7 ±4.7	4.5 (−0.2, 9.2)
***Median (IQR) ***	10 (10, 20)	20 (10, 20)	10 (10, 20)	
***Min – Max***	10–30	10–30	10–20	
**Visit 6**	**N=28**	**N=16**	**N=12**	
**No Presenteeism**	**11 (39.3)**	**5 (31.3)**	**6 (50.0)**	
**Presenteeism**	**17 (60.7)**	**11 (68.8)**	**6 (50.0)**	
***Missing***	**0 (0.0)**	**0 (0.0)**	**0 (0.0)**	
***Mean ±SD***	18.2 ±8.1	20.9 ±8.3	13.3 ±5.2	7.6 (−0.4, 15.6)
***Median (IQR) ***	20 (10, 20)	20 (20, 20)	10 (10, 20)	
***Min – Max***	10–40	10–40	10–20	

AS: Ankylosing spondylitis; CI: Confidence interval; IQR: Interquartile Range expressed as 25th–75th percentiles; nr-axSpA: Non-radiographic axial spondyloarthritis; SD: Standard deviation; WPAI-SHP: Work Productivity and Activity Impairment Specific Health Problem.

**Supplementary Table 8.** WPAI-SHP Work productivity loss scores (Percent overall work impairment due to problem–For Employed Patients only).

	**Overall**	**AS**	**nr-axSpA**	**Mean Difference (95% CI)**

**Visit 1**	**N=39**	**N=25**	**N=13**	
**No Productivity Loss**	**2 (5.1)**	**1 (4.0)**	**1 (7.1)**	
**Productivity Loss**	**37 (94.9)**	**24 (96.0)**	**13 (92.9)**	
***Missing***	**0 (0.0)**	**0 (0.0)**	**0 (0.0)**	
***Mean ±SD***	49.5 ±24.9	58.5 ±24.1	33.1 ±17	25.38 (11.5, 39.3)
***Median (IQR) ***	40 (30, 72)	56 (40, 81)	30 (20, 40)	
***Min – Max***	20–96	20–96	20–80	
**Visit 2**	**N=48**	**N=32**	**N=16**	
**No Productivity Loss**	**11 (22.9)**	**7 (21.9)**	**4 (25.0)**	
**Productivity Loss**	**37 (77.1)**	**25 (78.1)**	**12 (75.0)**	
***Missing***	**0**	**0**	**0**	
***Mean ±SD***	30.1 ±22.5	33.3 ±26.5	23.5 ±6.8	9.8 (−1.8, 21.3)
***Median (IQR) ***	20 (20, 30)	20 (20, 40)	20 (20, 30)	
***Min – Max***	10–100	10–100	10–33	
**Visit 3**	**N=48**	**N=29**	**N=19**	
**No Productivity Loss**	**13 (27.1)**	**7 (24.1)**	**6 (31.6)**	
**Productivity Loss**	**35 (72.9)**	**22 (75.9)**	**13 (68.4)**	
***Missing***	**0 (0.0)**	**0 (0.0)**	**0 (0.0)**	
***Mean ±SD***	23.6 ±13	25.5 ±14.3	20.4 ±10.1	5.1 (−4.0, 14.3)
***Median (IQR) ***	20 (10, 30)	20 (10, 40)	20 (10, 20)	
***Min – Max***	10–65	10–65	10–45	
**Visit 4**	**N=50**	**N=31**	**N=19**	
**No Productivity Loss**	**10 (20.0)**	**7 (22.6)**	**3 (15.8)**	
**Productivity Loss**	**40 (80.0)**	**24 (77.4)**	**16 (84.2)**	
***Missing***	**0 (0.0)**	**0 (0.0)**	**0 (0.0)**	
***Mean ±SD***	28.8 ±21.9	33.4 ±21	21.9 ±22	11.5 (−2.5, 25.5)
***Median (IQR)***	20 (10, 40)	30 (20, 40)	20 (10, 20)	
***Min – Max***	10–100	10–100	10–100	
**Visit 5**	**N=40**	**N=23**	**N=17**	
**No Productivity Loss**	**10 (25.0)**	**4 (17.4)**	**6 (35.3)**	
**Productivity Loss**	**30 (75.0)**	**19 (82.6)**	**11 (64.7)**	
***Missing***	**0 (0.0)**	**0 (0.0)**	**0 (0.0)**	
***Mean ±SD***	25.4 ±23	32.8 ±26.2	12.7 ±4.7	20.1 (7.2, 33.0)
***Median (IQR)***	18 (10, 26)	20 (10, 60)	10 (10, 20)	
***Min – Max***	10–100	10–100	10–20	
**Visit 6**	**N=28**	**N=16**	**N=12**	
**No Productivity Loss**	**11 (39.3)**	**5 (31.3)**	**6 (50.0)**	
**Productivity Loss**	**17 (60.7)**	**11 (68.8)**	**6 (50.0)**	
***Missing***	**0 (0.0)**	**0 (0.0)**	**0 (0.0)**	
***Mean ±SD***	30.3 ±20.7	39.5 ±20.1	13.3 ±5.2	26.2 (12.3, 40.1)
***Median (IQR)***	20 (10, 55)	40 (20, 60)	10 (10, 20)	
***Min – Max***	10–60	10–60	10–20	

AS: Ankylosing spondylitis; CI: Confidence interval; IQR: Interquartile Range expressed as 25th–75th percentiles; nr-axSpA: Non-radiographic axial spondyloarthritis; SD: Standard deviation; WPAI-SHP: Work Productivity and Activity Impairment Specific Health Problem.

**Supplementary Table 9.** WPAI-SHP Activity Impairment (Percent activity impairment due to problem).

	**Overall**	**AS**	**nr-axSpA**	**Mean Difference (95% CI)**

**Visit 1**	**N=85**	**N=56**	**N=29**	
**No Activity Impairment**	**3 (3.5)**	**2 (3.6)**	**1 (3.4)**	
**Activity Impairment**	**82 (96.5)**	**54 (96.4)**	**28 (96.6)**	
***Missing***	**0 (0.0)**	**0 (0.0)**	**0 (0.0)**	
***Mean ±SD***	55.6 ±21.5	60.7 ±20.6	45.7 ±19.9	15.0 (5.6, 24.5)
***Median (IQR) ***	50 (40, 80)	60 (50, 80)	40 (30, 60)	
***Min – Max***	20–90	20–90	20–80	
**Visit 2**	**N=85**	**N=56**	**N=29**	
**No Activity Impairment**	**11 (12.9)**	**5 (8.9)**	**6 (20.7)**	
**Activity Impairment**	**70 (82.4)**	**49 (87.5)**	**21 (72.4)**	
***Missing***	**4 (4.7)**	**2 (3.6)**	**2 (6.9)**	
***Mean ±SD***	26.1 ±12.4	28.4 ±13.4	21 ±7.7	7.4 (2.3, 12.5)
***Median (IQR) ***	20 (20, 30)	20 (20, 40)	20 (20, 20)	
***Min – Max***	10–70	10–70	10–40	
**Visit 3**	**N=82**	**N=55**	**N=27**	
**No Activity Impairment**	**15 (18.3)**	**8 (14.5)**	**7 (25.9)**	
**Activity Impairment**	**62 (75.6)**	**42 (76.4)**	**20 (74.1)**	
***Missing***	**5 (6.1)**	**5 (9.1)**	**0 (0.0)**	
***Mean ±SD***	24.7 ±12.5	27.6 ±13.6	18.5 ±6.7	9.1 (4.0, 14.3)
***Median (IQR)***	20 (20, 30)	30 (20, 40)	20 (10, 20)	
***Min – Max***	10–70	10–70	10–30	
**Visit 4**	**N=80**	**N=53**	**N=27**	
**No Activity Impairment**	**13 (16.3)**	**10 (18.9)**	**3 (11.1)**	
**Activity Impairment**	**42 (52.5)**	**24 (45.3)**	**18 (66.7)**	
***Missing***	**25 (31.3)**	**19 (35.8)**	**6 (22.2)**	
***Mean ±SD***	24 ±13.4	29.2 ±14.7	17.2 ±7.5	11.9 (4.9,
***Median (IQR ) ***	20 (10, 30)	25 (20, 40)	20 (10, 20)	
***Min – Max***	10–60	10–60	10–30	
**Visit 5**	**N=80**	**N=53**	**N=27**	
**No Activity Impairment**	**12 (15.0)**	**6 (11.3)**	**6 (22.2)**	
**Activity Impairment**	**28 (35.0)**	**17 (32.1)**	**11 (40.7)**	
***Missing***	**40 (50.0)**	**30 (56.6)**	**10 (37.0)**	
***Mean ±SD***	15 ±5.1	16.5 ±4.9	12.7 ±4.7	
***Median (IQR) ***	15 (10, 20)	20 (10, 20)	10 (10, 20)	
***Min – Max***	10–20	10–20	10–20	
**Visit 6**	**N=75**	**N=49**	**N=26**	
**No Activity Impairment**	**11 (14.7)**	**5 (10.2)**	**6 (23.1)**	
**Activity Impairment**	**17 (22.7)**	**11 (22.4)**	**6 (23.1)**	
***Missing***	**47 (62.7)**	**33 (67.3)**	**14 (53.8)**	
***Mean ±SD***	18.2 ±8.1	20.9 ±8.3	13.3 ±5.2	
***Median (IQR) ***	20 (10, 20)	20 (20, 20)	10 (10, 20)	
***Min – Max***	10–40	10–40	10–20	

AS: Ankylosing spondylitis; CI: Confidence interval; IQR: Interquartile Range expressed as 25th–75th percentiles; nr-axSpA: Non-radiographic axial spondyloarthritis; SD: Standard deviation; WPAI-SHP: Work Productivity and Activity Impairment Specific Health Problem.

**Supplementary Table 10.** Progression from nr-axSpA to AS during the study.

**Proportion of nr-axSpA patients who progressed to AS N(%)**	

	**N= 38**
Visit 2	4 (10.52)
Visit 3	0 (.)
Visit 4	0 (.)
Visit 5	0 (.)
Visit 6	0 (.)
**Time to progression from nr-axSpA to AS in days Patients Analysed**	38
**No. events**	4
Median	NR (NR- NR)
**Estimated hazard Ratios [HR (95% CI)]**	
Active Inflammation on MRI	1.31 (0.13, 12.81)
Age (per 1 year)	1.05 (0.92, 1.20)
Sex (Female Vs Male)	0.65 (0.18, 12.99)
Number of SpA features (per 1 unit)	0.87 (0.32, 2.41)
Good Response to NSAIDs (Yes Vs No)	0.75 (0.08, 6.92)
Current Treatment with NSAIDs (Yes Vs No)	0.56 (0.05, 6.62)

AS: Ankylosing Spondylitis; NR: not reached; nr-axSpA: Non-radiographic axial Spondyloarthritis; CI: 95% Confidence Interval.

**Supplementary Table 11.** Patient characteristics stratified by sex and axSpA Classification Criteria.

**Characteristic**	**AS**			**nr-axSpA**		

	**Male N=39**	**Female N=22**	p-value^*^	**Male N=15**	**Female N=14**	p-value^*^

**Mean age (years) (SD)**	42.7 (11.5)	46.8 (11.1)	0.199	39.9 (8.4)	42.8 (12.2)	0.451
**Mean symptom duration (months) (SD)**	101.0 (122.1)	98.5 (101.2)	0.562	42.1 (59.7)	88.4 (92.7)	0.290
**Mean time from diagnosis to baseline visit (months) (SD)**	3.4 (3.3)	2.9 (4.4)	0.142	3.2 (3.9)	2.2 (3.7)	0.363
**Number of SpA features†, mean (SD)**	4.5 (1.4)	4.3 (1.3)	0.616	4.0 (1.8)	2.9 (1.2)	0.123
Inflammatory back pain, n (%)	38 (97.4)	15 (68.2)	0.001	14 (100.0)	13 (86.7)	0.960
Psoriasis, n (%)	9 (23.1)	5 (22.7)	0.975	5 (35.7)	1 (6.7)	0.082
Arthritis, n (%)	29 (74.4)	15 (68.2)	0.605	10 (71.4)	11 (73.3)	0.474
Crohn’s disease or ulcerative colitis (IBD), n (%)	2 (5.1)	1 (4.5)	0.919	0 (0.0)	0 (0.0)	–
Enthesitis (heel), n (%)	31 (79.5)	13 (59.1)	0.088	8 (57.1)	5 (33.3)	0.340
Good Response to NSAIDs, n (%)	15 (38.5)	9 (40.9)	0.851	5 (35.7)	3 (20.0)	0.747
Uveitis, n (%)	1 (2.6)	0 (0.0)	0.449	2 (14.3)	1 (6.7)	0.584
Family History of SpA, n (%)	3 (7.7)	1 (4.5)	0.634	0 (0.0)	0 (0.0)	–
Dactylitis, n (%)	1 (2.6)	1 (4.5)	0.676	3 (21.4)	0 (0.0)	0.077
HLA-B27 positive‡, n (%)	23 (59.0)	3 (13.6)	0.001	3 (21.4)	2 (13.3)	0.684
Elevated CRP, n (%)	25 (64.1)	10 (45.5)	0.157	10 (71.4)	4 (26.7)	0.040
**Mean CRP, mg/L, (SD)**	34.4 (38.9)	24.2 (22.8)	0.436	12.2 (16.7)	9.2 (11.1)	0.867
**Mean ASDAS-CRP/ESR, (SD)**	4 (0.7)	3.6 (0.9)	0.185	3.2 (0.8)	2.9 (0.9)	0.616
**Mean BASDAI, (SD)**	6.2(1.5)	5.5(2)	0.301	4.9(1.9)	4.5(2)	0.644
**Mean BASFI, (SD)**	5.7(1.8)	5.2(2.1)	0.642	3.4(1.3)	3.4(1.8)	>0.99
Sacroiliitis on MRI, n(%)			0.787			0.588
Active inflammation on MRI highly suggestive of sacroiliitis associated with SpA, n (%)	2 (5.1)	1 (4.5)		6 (42.9)	7 (46.7)	
Definite radiographic sacroiliitis according to modified New York criteria, n (%)	36 (92.3)	14 (63.6)		9 (64.3)	7 (46.7)	
Both, n (%)	1 (2.6)	1 (4.5)		0 (0.0)	0 (0.0)	

* For the comparison of male vs female.

† SpA features included in the ASAS classification criteria for axSpA excluding imaging.

‡ Based on patients with HLA-B27 assessed.

AS: Ankylosing Spondylitis; ASDAS-CRP/ESR: Ankylosing Spondylitis Disease Activity Score containing CRP or ESR when CRP is not available; BASDAI: Bath Ankylosing Spondylitis Disease Activity Index; BASFI: Bath Ankylosing Spondylitis Functional Index; CRP: C-reactive protein; ESR: Erythrocyte sedimentation rate; HLA-B27: human leukocyte antigen B27; IBD: inflammatory bowel disease; MRI: Magnetic resonance imaging; nr-axSpA: non-radiographic axial Spondyloarthritis; NSAID: non-steroidal anti-inflammatory drugs; SD: standard deviation; SpA; Spondyloarthritis.

**Supplementary Table 12.** Patient characteristics stratified by sex and axSpA classification criteria.

	**AS**						**nr-axSpA**					

	**TNF inhibitor use**			**No TNF inhibitor use**			**TNF inhibitor use**			**No TNF inhibitor use**		

**Characteristic**	**Male N=34**	**Female N=9**	p-value^*^	**Male N=5**	**Female N=8**	p-value^*^	**Male N=10**	**Female N=9**	p-value^*^	**Male N=5**	**Female N=5**	p-value^*^

**Mean age (years) (SD)**	41.3 (11.3)	48.3 (7.2)	**0.031**	51.8 (9.3)	45.1 (14.8)	0.106	43.2 (6.5)	46.2 (11.4)	0.497	33.2 (8.2)	36.6 (12.2)	0.690
**Mean symptom duration (months) (SD)**	86.0 (111.9)	99.1 (63.2)	0.177	203.0 (153.0)	97.8 (137.2)	0.092	57.8 (68.5)	121.8 (99.9)	0.278	10.6 (7.8)	28.2 (30.6)	0.841
**Mean time from diagnosis to baseline visit (months) (SD)**	3.3 (3.2)	3.4 (4.9)	0.508	3.8 (4.2)	2.3 (4.2)	0.174	2.5 (2.2)	3.0 (4.2)	0.780	4.6 (6.3)	0.3 (0.5)	0.556
**Number of SpA†** **features, mean (SD)**	4.6 (1.4)	4.4 (1.3)	0.918	4.4 (1.1)	4.1 (1.3)	0.833	3.9 (2.0)	2.3 (0.5)	0.113	4.2 (1.6)	3.8 (1.5)	0.690
Inflammatory back pain, n (%)	33 (97.1)	9 (100.0)	0.603	5 (100.0)	6 (75.0)	0.224	10 (100.0)	9 (100.0)	–	4 (80.0)	4 (80.0)	>0.99
Psoriasis, n (%)	7 (20.6)	2 (22.2)	0.915	2 (40.0)	3 (37.5)	0.928	3 (30.0)	0 (0.0)	0.073	2 (40.0)	1 (20.0)	0.49
Arthritis, n (%)	24 (70.6)	9 (100.0)	0.063	5 (100.0)	6 (75.0)	0.224	7 (70.0)	7 (77.8)	0.701	3 (60.0)	4 (80.0)	0.49
Crohn’s disease or ulcerative colitis (IBD), n (%)	2 (5.9)	1 (11.1)	0.584	0 (0.0)	0 (0.0)	–	0 (0.0)	0 (0.0)	–	0 (0.0)	0 (0.0)	–
Enthesitis (heel), n (%)	27 (79.4)	7 (77.8)	0.915	4 (80.0)	6 (75.0)	0.835	4 (40.0)	1 (11.1)	0.153	4 (80.0)	4 (80.0)	>0.89
Good Response to NSAIDs, n (%)	14 (41.2)	4 (44.4)	0.860	1 (20.0)	5 (62.5)	0.135	2 (20.0)	0 (0.0)	0.156	3 (60.0)	3 (60.0)	>0.99
Uveitis, n (%)	1 (2.9)	0 (0.0)	0.603	0 (0.0)	0 (0.0)	–	1 (10.0)	1 (11.1)	0.937	1 (20.0)	0 (0.0)	0.292
Family History of SpA, n (%)	3 (8.8)	1 (11.1)	0.836	0 (0.0)	0 (0.0)	–	0 (0.0)	0 (0.0)	–	0 (0.0)	0 (0.0)	–
Dactylitis, n (%)	1 (2.9)	0 (0.0)	0.603	0 (0.0)	1 (12.5)	0.411	2 (20.0)	0 (0.0)	0.156	1 (20.0)	0 (0.0)	0.292
HLA-B27 positive, n (%)	**21 (61.8)**	**2 (22.2)**	**0.034**	2 (40.0)	1 (12.5)	0.252	3 (30.0)	2 (22.2)	0.701	0 (0.0)	0 (0.0)	–
Elevated CRP, n (%)	22 (64.7)	5 (55.6)	0.614	3 (60.0)	5 (62.5)	0.928	7 (70.0)	1 (11.1)	0.009	3 (60.0)	3 (60.0)	>0.99
**Mean CRP, mg/L (SD)**	37.3(40.1)	25.9(22.3)	0.589	21(33.4)	22.5(24.9)	0.807	24.8(20.2)	11.4(18.7)	0.127	2.8(2.5)	7.8(5.8)	0.169
**Mean ASDAS-CRP/ESR (SD)**	4.0 (0.6)	3.7 (0.5)	0.178	3.9 (1.4)	3.5 (1.2)	0.465	3.2 (0.8)	2.9 (0.8)	0.659	3.2 (0.8)	3.0 (1.2)	>0.99
**Mean BASDAI (SD)**	6 (1.4)	5.2 (2.1)	0.309	7.3 (1.8)	5.9 (1.9)	0.306	4.2 (1.7)	3.6 (1.7)	0.647	6.6 (1.1)	6 (1.8)	0.462
**Mean BASFI (SD)**	5.5 (1.5)	5.2 (1.9)	0.866	6.8 (3.4)	5.3 (2.4)	0.222	3.7 (1.3)	3.3 (1.6)	>0.99	3.0 (1.2)	3.5 (2.2)	>0.99
**Sacroiliitis on MRI, n (%)**			0.470			0.715			0.906			0.292
Active inflammation on MRI highly												
suggestive of sacroiliitis associated with SpA, n (%)	1 (2.9)	0 (0.0)		1 (20.0)	1 (12.5)		2 (20.0)	2 (22.2)		4 (80.0)	5 (100.0)	
Definite radiographic sacroiliitis according to modified New York criteria, n (%)	32 (94.1)	7 (77.8)		4 (80.0)	7 (87.5)		8 (80.0)	7 (77.8)		1 (20.0)	0 (0.0)	
Both, n (%)	1 (2.9)	1 (11.1)		0 (0.0)	0 (0.0)		0 (0.0)	0 (0.0)		0 (0.0)	0 (0.0)	

* For the comparison of male vs female.

† SpA features included in the ASAS classification criteria for axSpA excluding imaging.

‡ Based on patients with HLA-B27 assessed.

AS: Ankylosing Spondylitis; ASDAS-CRP/ESR: Ankylosing Spondylitis Disease Activity Score containing CRP or ESR when CRP is not available; BASDAI: Bath Ankylosing Spondylitis Disease Activity Index; BASFI: Bath Ankylosing Spondylitis Functional Index; CRP: C-reactive protein; ESR: Erythrocyte sedimentation rate; HLA-B27: human leukocyte antigen B27; IBD: inflammatory bowel disease; MRI: Magnetic resonance imaging; nr-axSpA: non-radiographic axial Spondyloarthritis; NSAID: non-steroidal anti-inflammatory drugs; SD: standard deviation; SpA; Spondyloarthritis.

**Supplementary Table 13.** ASDAS-CRP/ESR category results stratified by sex and axSpA Classification Criteria.

**Visit & Category**	**AS**			**Nr-axSpA**		
	
	**Male N=38**	**Female N=22**	p-value^*^	**Male N=24**	**Female N=16**	p-value^*^

**Baseline**	**N=34**	**N=14**		**N=13**	**N=14**	
Inactive disease	0 (0.0)	0 (0.0)	–	0 (0.0)	2 (14.3)	0.157
Low disease activity	0 (0.0)	0 (0.0)	–	1 (7.7)	0 (0.0)	0.290
High disease activity	7 (20.6)	5 (35.7)	0.271	8 (61.5)	9 (64.3)	0.883
Very high disease activity	27 (79.4)	9 (64.3)	0.271	4 (30.8)	3 (21.4)	0.580
**Visit 2**	**N=36**	**N=17**		**N=13**	**N=13**	
Inactive disease	5 (13.9)	4 (23.5)	0.383	7 (53.8)	5 (38.5)	0.343
Low disease activity	13 (36.1)	4 (23.5)	0.360	0 (0.0)	2 (15.4)	0.157
High disease activity	17 (47.2)	8 (47.1)	0.991	6 (46.2)	6 (46.2)	>0.99
Very high disease activity	1 (2.8)	1 (5.9)	0.580	0 (0.0)	0 (0.0)	–
**Visit 3**	**N=36**	**N=15**		**N=13**	**N=13**	
Inactive disease	9 (25.0)	2 (13.3)	0.356	6 (46.2)	4 (30.8)	0.420
Low disease activity	14 (38.9)	8 (53.3)	0.343	4 (30.8)	5 (38.5)	0.680
High disease activity	13 (36.1)	4 (26.7)	0.514	3 (23.1)	4 (30.8)	0.658
Very high disease activity	0 (0.0)	1 (6.7)	0.118	0 (0.0)	0 (0.0)	–
**Visit 4**	**N=37**	**N=15**		**N=13**	**N=13**	
Inactive disease	6 (16.2)	3 (20.0)	0.744	4 (30.8)	9 (69.2)	0.05
Low disease activity	17 (45.9)	10 (66.7)	0.175	9 (69.2)	3 (23.1)	0.018
High disease activity	14 (37.8)	2 (13.3)	0.083	0 (0.0)	1 (7.7)	0.308
Very high disease activity	0 (0.0)	0 (0.0)	–	0 (0.0)	0 (0.0)	–
**Visit 5**	**N=33**	**N=15**		**N=13**	**N=12**	
Inactive disease	6 (18.2)	4 (26.7)	0.502	5 (38.5)	7 (58.3)	0.320
Low disease activity	23 (69.7)	7 (46.7)	0.127	7 (53.8)	3 (25.0)	0.141
High disease activity	4 (12.1)	4 (26.7)	0.210	1 (7.7)	2 (16.7)	0.490
Very high disease activity	0 (0.0)	0 (0.0)	–	0 (0.0)	0 (0.0)	–
**Visit 6**	**N=33**	**N=14**		**N=13**	**N=8**	
Inactive disease	9 (27.3)	3 (21.4)	0.590	9 (69.2)	2 (25.0)	0.049
Low disease activity	19 (57.6)	7 (50.0)	0.482	3 (23.1)	4 (50.0)	0.204
High disease activity	5 (15.2)	4 (28.6)	0.343	1 (7.7)	2 (25.0)	0.271
Very high disease activity	0 (0.0)	0 (0.0)	–	0 (0.0)	0 (0.0)	–

ASDAS-CRP/ESR: Ankylosing Spondylitis Disease Activity Score containing CRP or ESR when CRP is not available; AS: Ankylosing Spondylitis; BASDAI; Bath Ankylosing Spondylitis Disease Activity Index; nr-axSpA; non-radiographic axial Spondyloarthritis; SpA; Spondyloarthritis.

Disease activity states were assigned using the ASDAS cut-off levels: inactive disease (ASDAS <1.3); low disease activity (1.3 ≤ASDAS <2.1); high disease activity (2.1 ≤ASDAS ≤3.5); and very high disease activity (ASDAS >3.5).

**Supplementary Table 14.** BASDAI and BASFI results stratified by sex and SpA classification criteria.

	**AS**			**nr-axSpA**		

**BASDAI**						

**Visit**	**Male N=38**	**Female N=22**	p-value^*^	**Male N=24**	**Female N=16**	p-value^*^

**Baseline, N**	**N=39**	**N=17**		**N=14**	**N=14**	
Mean (SD)	6.2 (1.5)	5.5 (2)	0.301	4.9 (1.9)	4.5 (2)	0.644
**Visit 2, N**	**N=37**	**N=17**		**N=13**	**N=13**	
Mean (SD)	2.6 (1.6)	2.5 (1.2)	0.794	1.6 (1.6)	1.9 (0.9)	0.372
**Visit 3, N**	**N=36**	**N=15**		**N=13**	**N=13**	
Mean (SD)	2.2 (1.6)	1.9 (1.5)	0.487	1.2 (1.1)	1.7 (1)	0.339
**Visit 4, N**	**N=37**	**N=16**		**N=13**	**N=13**	
Mean (SD)	2.6 (1.8)	1.9 (1.3)	0.130	1.4 (0.9)	1.4 (0.8)	0.875
**Visit 5, N**	**N=33**	**N=15**		**N=13**	**N=12**	
Mean (SD)	1.7 (1.1)	1.6 (1.1)	0.553	0.9 (0.8)	1.1 (0.6)	0.418
**Visit 6, N**	**N=33**	**N=14**		**N=13**	**N=8**	
Mean (SD)	1.8 (1.1)	2.2 (1.5)	0.656	0.7 (0.8)	1.9 (1.3)	0.021

**BASFI**						

**Baseline, N**	**N=38**	**N=17**		**N=15**	**N=13**	
Mean (SD)	5.7 (1.8)	5.2 (2.1)	0.642	3.4 (1.3)	3.4 (1.8)	>0.99
**Visit 2, N**	**N=37**	**N=17**		**N=14**	**N=12**	
Mean (SD)	2.6 (1.6)	2.3 (1.2)	0.830	1.3 (1.2)	1.7 (1)	0.252
**Visit 3, N**	**N=35**	**N=15**		**N=14**	**N=12**	
Mean (SD)	2.2 (1.5)	1.8 (1.2)	0.611	1.1 (1.1)	1.4 (0.7)	0.527
**Visit 4, N**	**N=35**	**N=16**		**N=14**	**N=12**	
Mean (SD)	2.6 (2.1)	1.9 (1.4)	0.226	0.9 (0.8)	1 (0.6)	0.631
**Visit 5, N**	**N=32**	**N=15**		**N=14**	**N=11**	
Mean (SD)	1.9 (1.5)	1.4(1.1)	0.231	0.7 (0.7)	1 (0.8)	0.401
**Visit 6, N**	**N=32**	**N=14**		**N=13**	**N=7**	
Mean (SD)	1.9 (1.3)	2 (1.3)	0.904	0.7 (0.9)	1.7 (1.2)	0.097

AS: Ankylosing Spondylitis; BASDAI: Bath Ankylosing Spondylitis Disease Activity Index; BASFI: Bath Ankylosing Spondylitis Functional Index; nraxSpA: non-radiographic axial Spondyloarthritis; SpA: Spondyloarthritis; SD: standard deviation.

* For the comparison of male vs female.

## OPEN DATA SHARING STATEMENT

AbbVie is committed to responsible data sharing regarding the clinical trials we sponsor. This includes access to anonymised, individual, and trial-level data (analysis data sets), as well as other information (eg, protocols, clinical study reports, or analysis plans), as long as the trials are not part of an ongoing or planned regulatory submission. This includes requests for clinical trial data for unlicensed products and indications.

These clinical trial data can be requested by any qualified researchers who engage in rigorous, independent, scientific research, and will be provided following review and approval of a research proposal, Statistical Analysis Plan (SAP), and execution of a Data Sharing Agreement (DSA). Data requests can be submitted at any time after approval in the US and Europe and after acceptance of this manuscript for publication. The data will be accessible for 12 months, with possible extensions considered. For more information on the process or to submit a request, visit the following link: https://vivli.org/ourmember/abbvie/
